# Dendritic Nano‐Based Slippery Coating by Synergistic Mechanical and Electrostatic Interactions with Persistent and Exceptional Combats Thrombosis

**DOI:** 10.1002/advs.202521672

**Published:** 2026-01-29

**Authors:** Shu Zhang, Yao Shen, Juan Liu, Qing Zeng, Yunze Ma, Shuping Chen, Taiyu Nan, Xiaoying Qiu, Jiayi Yu, Tao Fan, Guozhi Huang, Jihua Zou, Chengduan Yang

**Affiliations:** ^1^ Center of Rehabilitation Medicine, Zhujiang Hospital, Southern Medical University School of Rehabilitation Sciences, Southern Medical University Key Laboratory of Brain Function Detection and Neuromodulation Intelligent Rehabilitation of Guangdong Higher Education Institutes Guangdong Engineering Technology Research Center for Brain Function Detection and Neuromodulation Rehabilitation Guangzhou China; ^2^ Rice Research Institute Guang Dong Academy of Agricultural Sciences Guangzhou China

**Keywords:** anti‐biofouling and anti‐thrombotic, dendritic nano‐based slippery coating (DNSC), mechanical interlock‐electrostatic anchoring, persistent and exceptional, slippery

## Abstract

Implantable medical devices face critical failure risks due to biofouling and thrombosis. A major challenge is developing coatings that combine long‐term mechanical stability with robust anti‐biofouling efficacy under dynamic vascular conditions. To address this, we introduce a novel dendritic nano‐based slippery coating (DNSC), fabricated by encapsulating carboxyl silicone oil within amino dendritic silica nanoparticles and co‐embedding them in an epoxy resin matrix. The dendritic architecture enhances nanoparticle dispersion in the matrix and establishes mechanical interlock. Coupled with electrostatic interactions between amine and carboxyl groups, this design ensures stable lubricant immobilization, improving mechanical durability while providing exceptional slipperiness. Under simulated blood flow, DNSC demonstrated unprecedented and sustained resistance to protein, bacterial, cellular, and platelet adhesion for over 15 days. Mechanistic studies confirmed that anti‐adhesion arises from physical slippage rather than bioactive release. Besides, in vitro and in vivo evaluations showed significant thrombosis inhibition without notable inflammation or tissue damage, confirming excellent biocompatibility. Through synergistic innovation in material architecture and interfacial engineering, this work successfully resolves the longstanding trade‐off between mechanical robustness and surface slippery. The proposed DNSC offers a promising surface modification strategy for blood‐contacting devices, integrating durability, anti‐fouling performance, and biosafety to enhance device reliability and longevity.

## Graphical abstract

A novel dendritic nano‐based slippery coating (DNSC), fabricated by encapsulating carboxyl silicone oil within amino dendritic silica nanoparticles and co‐embedding them in an epoxy resin matrix. The dendritic architecture enhances nanoparticle dispersion in the matrix and establishes mechanical interlock. Coupled with electrostatic interactions between amine and carboxyl groups, this design ensures stable lubricant immobilization, improving mechanical durability while providing exceptional slipperiness. Under simulated blood flow, DNSC demonstrated unprecedented and sustained resistance to protein, bacterial, cellular, and platelet adhesion for over 15 days. The proposed DNSC offers a promising surface modification strategy for blood‐contacting devices, integrating durability, anti‐fouling performance, and biosafety to enhance device reliability and longevity.

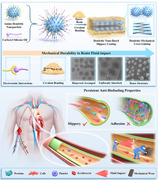

## 1 Introduction

Implantable medical devices, such as artificial hearts [1], pacemakers [2], and central venous catheters [3], require anti‐biofouling surfaces with long‐term stability and biocompatibility to ensure clinical efficacy and patient safety. Such surfaces are critical for preventing thrombosis and preserving device functionality during prolonged use. Although heparinization is a widely employed strategy for localized thrombosis inhibition [4, 5], its drug‐release mechanism presents inherent limitations. Heparin immobilized on device surfaces tends to gradually leach under physiological blood flow, leading to diminished anticoagulant activity over time. Furthermore, this release process may trigger adverse effects, including heparin‐induced thrombocytopenia, hemorrhagic complications, and paradoxical thrombotic events [6, 7, 8]. These challenges underscore the inadequacy of release‐dependent coatings for sustained, long‐term implantation applications.

Recently, various surface modification strategies have been developed, including superhydrophilic [3], superhydrophobic [9], and slippery liquid‐infused porous surfaces (SLIPS or LIS) [10, 11]. Conventional superhydrophobic surfaces, while exhibiting excellent liquid repellency, often suffer from inherent drawbacks. Their performance critically depends on fragile hierarchical micro/nanostructures that are susceptible to mechanical abrasion or chemical degradation, leading to irreversible loss of superhydrophobicity and fouling resistance [12, 13]. In contrast, SLIPS/LIS have attracted considerable attention due to a distinct mechanism that leverages a physically smooth liquid lubricant layer to achieve outstanding slipperiness and interfacial chemical stability. This approach has shown promise for robust anti‐fouling and anti‐corrosion applications, with recent research focusing on enhancing their mechanical durability and multifunctionality [14]. For example, Yin et al. demonstrated effective inhibition of bacterial adhesion and blood coagulation by modifying surfaces with fluorosilane and immobilizing perfluorocarbon lubricants via fluorine–fluorine interactions [15]. However, such lubricant layers are typically anchored through porous substrates or physical adsorption mechanisms (e.g., van der Waals forces) [16, 17, 18], rendering them prone to depletion under fluid shear stress and compromising the long‐term functionality of the coating. Although low‐surface‐energy, “liquid‐like” polymer brushes constructed through covalent grafting can enhance interfacial stability, their highly cross‐linked networks often restrict molecular chain mobility while improving mechanical properties, consequently compromising both surface slipperiness and anti‐fouling efficacy [19, 20]. Fundamentally, the design of anti‐fouling coatings faces an intrinsic trade‐off: while low‐surface‐energy materials—such as fluorinated compounds and siloxanes—exhibit strong resistance to fouling [14, 21, 22], their weak interfacial adhesion, leads to inadequate mechanical durability and abrasion resistance [23]. In contrast, highly cross‐linked polymer networks, despite offering superior mechanical strength, generally possess high surface energy, which increases susceptibility to biofouling and impedes the attainment of a slippery surface [24, 25]. Consequently, the development of anti‐fouling coatings that simultaneously achieve durable stability and excellent slipperiness remains a significant challenge.

Recent studies have attempted to synergistically enhance the anti‐wear and anti‐fouling properties of coatings by incorporating inorganic fillers with low‐surface‐energy polymers. For example, Zhang et al. developed a mechanically robust anti‐fouling coating using diamino‐terminated polydimethylsiloxane as a cross‐linking agent to bridge epoxy‐modified hydrophobic silica and an epoxy‐silicone prepolymer [14]. Similarly, several studies have fabricated “solid‐like” slippery coatings by embedding nanoparticles—functioning as silicone oil reservoirs—within an epoxy resin matrix, thereby achieving durable anti‐fouling protection for devices [26, 27, 28]. Although inorganic nano‐reinforcement strategies hold promise for long‐term anti‐fouling performance [29], their application in anti‐thrombogenic coatings for blood‐contacting devices remains hindered by several critical challenges. First, functional nanoparticles are prone to aggregation due to van der Waals interactions, which may induce structural defects and interfacial inhomogeneity, thereby compromising coating stability, limiting lubricant retention, and ultimately diminishing anti‐fouling efficacy [30, 31, 32]. Second, most current nano‐based composite coatings are designed for macroscopic functionalities, such as liquid repellency or ice prevention [26, 27, 28], leaving the mechanisms and effectiveness of these coatings against biofouling—such as protein, bacterial, and cells adhesion—poorly understood. Finally, whether such coatings can achieve long‐term safe anticoagulant effects still requires in‐depth verification.

In the study, we developed a novel dendritic nano‐based slippery coating (DNSC) on the surface of medical catheters (DNSC‐MC). The coating comprises amino‐functionalized dendritic nanoparticles that immobilize carboxyl‐functionalized silicone oil within an epoxy resin matrix. The composite architecture enables sustained and robust antithrombotic performance through the synergistic effects of mechanical interlocking and electrostatic interactions. The dendritic morphology of nanoparticles enhances dispersibility and facilitates strong mechanical anchoring. Together with the electrostatic interaction between amino and carboxyl groups, the structure effectively stabilizes the lubricant phase, thereby concurrently improving the coating's mechanical integrity and slipperiness. Under simulated blood flow conditions (shear rate ∼ 1750 s^−^^1^), DNSC‐MC demonstrated an unprecedented resistance to the adhesion of proteins, bacteria, mammalian cells, and platelets for more than 15 days. Integrated analyses including slip performance testing, biological activity assessment, and cellular morphology evaluation confirm that the antifouling properties arise from the coating's superior slipperiness and demonstrate excellent biocompatibility. Moreover, DNSC‐MC significantly suppressed thrombus formation in both in vitro and in vivo circulation models over prolonged durations, without eliciting inflammatory responses or causing organ or tissue damage. The work presents a new surface functionalization strategy that integrates biosafety, strong resistance to bioadhesion, and enhanced durability through a “slippery” mechanism. The approach offers a promising foundation for the development of anti‐biofouling and antithrombotic implantable biomedical devices and introduces a novel paradigm for the application of nano‐based slippery coatings in biomedical antifouling applications.

## 2 Results and Discussion

### 2.1 Design and Preparation of the Dendritic Nano‐Based Slippery Coating

**FIGURE 1:**
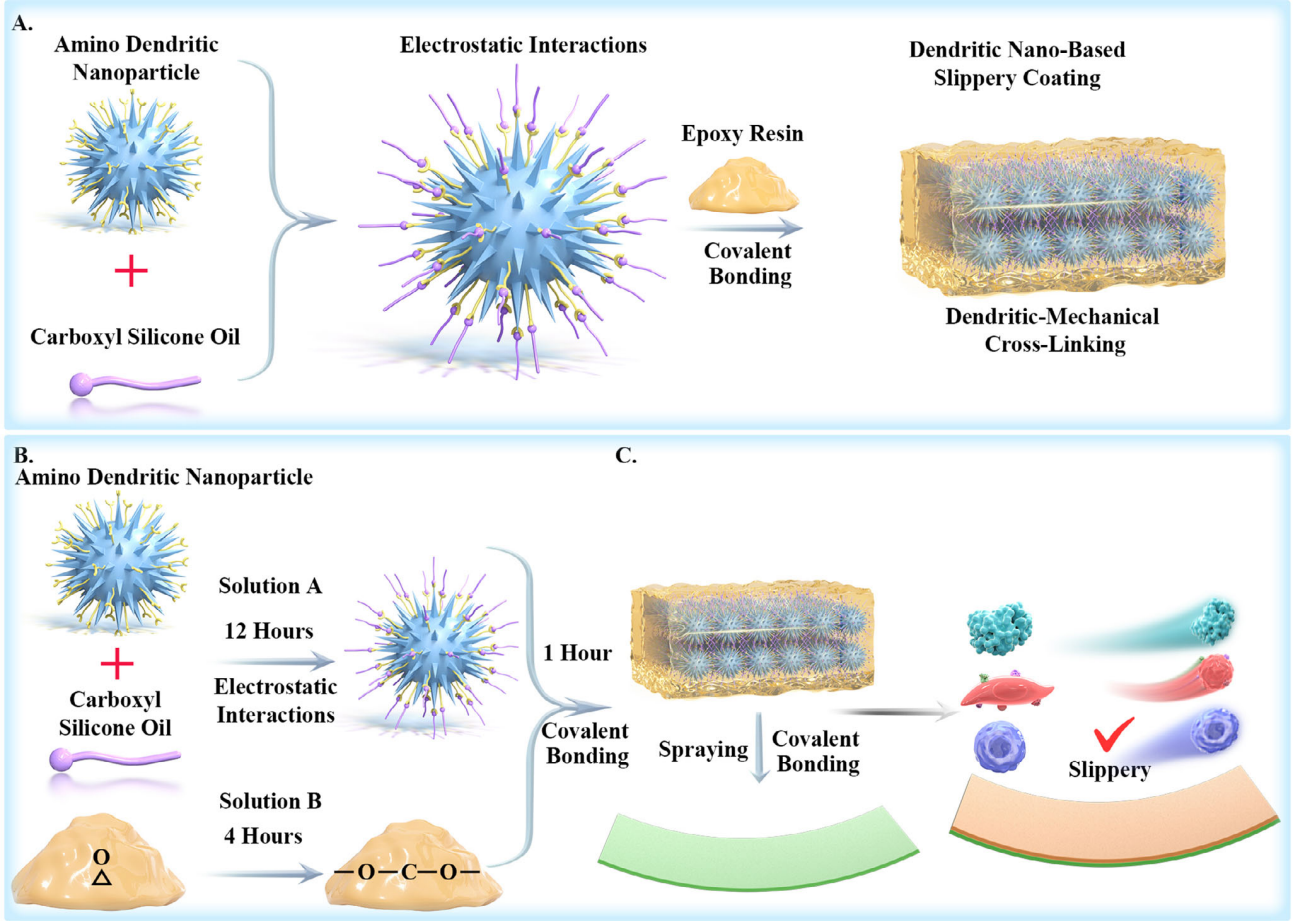
Design and Preparation of the dendritic nano‐based slippery coating. (A,B) The schematic diagram and fabrication procedure of uniformly dispersed amino dendritic nanoparticles capturing ─COOH silicone oil through electrostatic interaction to form a uniform interlocked cross‐linked particle system. Then covalently cross‐linked with epoxy resin to form a dense framework composite cross‐linked system. (C) The dendritic nano‐based slippery coating solution was evenly sprayed on the surface, and the covalent cross‐linking reaction was carried out. Eventually, this mechanically stable and wear‐resistant slippery surface achieves mechanical durability to resist fluid impact, and persistent anti‐biofouling properties.

The design strategy of the dendritic nano‐based slippery coating (DNSC) we designed includes three key elements: (1) Leveraging the steric hindrance effect of nanospikes, the surface amino‐functionalized dendritic nanoparticles achieve an exceptionally stable dispersion and robust mechanical interlocking. Then, through collective effects of molecular forces — including electrostatic interactions and van der Waals forces with carboxyl (─COOH) silicone oil — these nanoparticles effectively capture the lubricant, collectively endowing the material with mechanical stability and outstanding “slippery”. (2) Via covalent crosslinking, oil‐storing dendritic particles are embedded both within and on the surface of epoxy resin, forming a homogeneous and structurally dense composite crosslinking system. This significantly enhances the mechanical strength and wear resistance of the composite coating while preserving its “slippery” function. (3) By means of the spraying process, DNSC solution is coated on the surface of hydroxyl‐rich substrates (such as glass, plasma‐treated catheter, etc.). Then, the coating forms further covalent cross‐linking between the epoxy group and the substrate, and cures spontaneously, which significantly enhances the interfacial adhesion and exceeds simple physical anchoring. Figure 1A schematically illustrates the structure of the DNSC with mechanical stability and high “slippery”.

**FIGURE 2:**
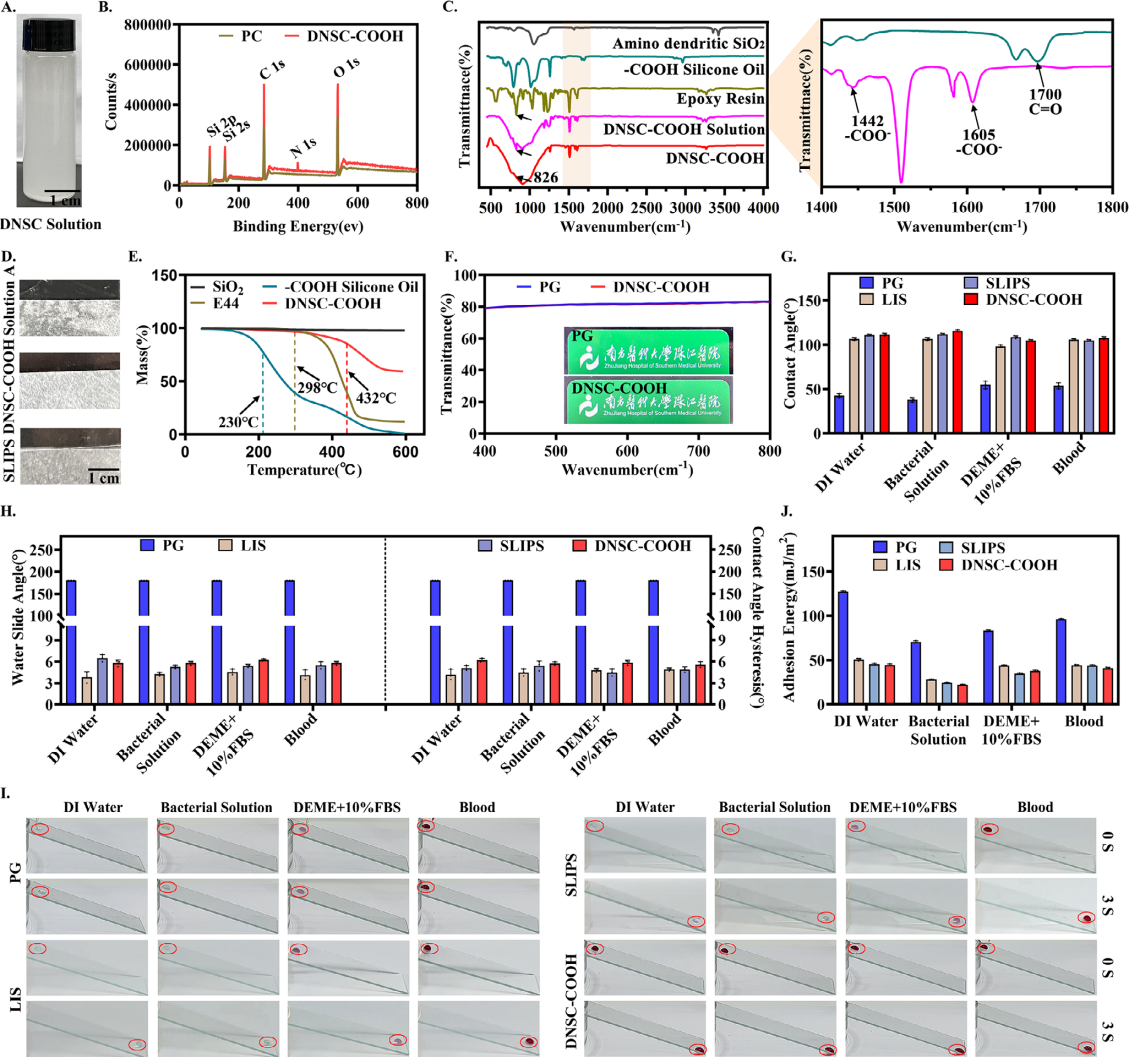
Characterization of the dendritic nano‐based slippery coating. (A) The synthetic DNSC solution. (B) The elemental composition and content on the catheter before and after DNSC‐COOH modification were detected by XPS. (C) FTIR test results of different substances in DNSC‐COOH, including amino nanoparticles, ─COOH silicone oil, epoxy resin, DNSC‐COOH solution and DNSC‐COOH modified catheter surface. (D) On solution A coating, DNSC‐COOH and typical SLIPS surfaces, the free lubricant on different surfaces was observed using oil testing paper. (E) TGA results. (F) Transmission spectroscopy test and clarity comparison results of PG and DNSC‐COOH (about 10 µm) surfaces. (G,H) Statistical results of CA, SA, and CAH of different types of water‐based droplets on different surfaces (PG, LIS, SLIPS, and DNSC‐COOH). (I) Observe the differences in the dynamic sliding performance of different surfaces on different droplets under an inclination angle of approximately 30° in 3 s. (J) Statistics of theoretical calculation results of adhesion energy of different surfaces to different droplets. Error bar represents the mean ± SD. *n* = 3, averaged.

The preparation of DNSC was completed in two steps (Figure 1B,C). First, preparation of the DNSC solution. Initially, through collective effects of molecular forces such as electrostatic interactions and van der Waals forces, the abnormally dispersed amino functionalized dendritic nanoparticles can capture silicone oil with different terminations and store them — either stably or unstably — on their surface. Subsequently, through the covalent crosslinking reaction, the oil‐storing nanoparticles became uniformly distributed on and inside the epoxy resin, yielding a translucent composite slippery coating solution (Figure 2A). Second, the composite slippery coating solution was uniformly applied onto the plasma‐treated substrate using the spraying technique. The epoxy group and hydroxyl group can be covalently cross‐linked and self‐cured to form a mechanically robust and wear‐resistant slippery surface to achieve long‐term resistance against various biological pollutants and thrombosis formation.

### 2.2 Characterization of the Dendritic Nano‐Based Slippery Coating

X‐ray photoelectron spectroscopy (XPS) was employed to verify the successful deposition of DNSC, which was constructed by ─COOH‐terminated silicone oil (DNSC‐COOH). Compared with the surface of unmodified primitive polyvinyl chloride (PVC) catheter (PC), where only a trace amount of silicon signal possibly originating from processing additives was detected, Si 2p (102 ev), Si 2s (153 ev), and N 1s (398 ev) peaks were significantly enhanced on the surface of DNSC‐COOH modified catheter (DNSC‐MC) (Figure 2B). It is notable that the binding energy of the O 1s main peak of the DNSC‐COOH system increased from 531.75 to 532.38ev (Figure S1), suggesting that oxygen atoms might have interfuncted with stronger groups [33]. In the Fourier transform infrared (FTIR) spectroscopy results (Figure 2C), the DNSC‐COOH solution exhibited a disappearance of the stretching‐vibration absorption peak at 1700 cm^−^^1^ compared to pure ─COOH silicone oil, while new absorption peaks emerged at 1442 and 1605 cm−^1^. This change was mainly caused by the loss of a proton from carboxyl groups to form ─COO^−^, which confirmed the electrostatic interactions between ─COOH silicone oil and amino nanoparticles [34, 35]. Furthermore, compared to the epoxy resin, the epoxy characteristic peak (826 cm^−1^) gradually decreased in both the DNSC‐COOH solution and its modified surface, indicating that the epoxy resin acts as a coupling agent to immobilize oil‐storing particles onto the substrate surface via covalent crosslinking. The state of lubricant within the DNSC‐COOH was further investigated by using oil test paper and thermogravimetric analysis (TGA). The DNSC‐COOH was covered with oil test paper, and the solution A (formed by ─COOH silicone oil and amino‐functionalized nanoparticles) and SLIPS coating were used as controls. The results (Figure 2D) showed that the test paper remained dry and clean on the DNSC‐COOH surface, indicating no residual liquid oil. In contrast, the test paper was wetted on surfaces modified with solution A coating (prior to epoxy resin addition), similar to the SLIPS, suggesting that solution A coating contained liquid‐flowing oil. This mechanism likely involves amino‐functionalized nanoparticles first binding to silicone oil molecules through electrostatic interactions, then further followed by van der Waals forces capturing the liquid‐flowing oil. However, with the incorporation of epoxy resin, the nanoparticles that captured silicone oil were embedded into its surface and interior, thereby inhibiting its macroscopic migration and leakage. As shown in Figure 2E, the TGA results showed that compared with ─COOH silicone oil, DNSC‐COOH's initial decomposition temperature was increased to 432°C, which further proved that the electrostatic interactions, van der Waals forces and other molecular forces between amino functionalized nanoparticles and ─COOH silicone oil were generated, which improves the thermal stability. Furthermore, the impact of DNSC‐COOH on optical transmittance was also investigated. UV–vis transmission spectroscopy (Figure 2F) showed that within the visible light range, the transmittance of DNSC (about 10 µm) reached approximately 80%, nearly identical to that of primitive glass (PG, about 81%). Meanwhile, the logos and text covered by DNSC‐COOH modified glass remained clearly visible without significant deterioration in clarity. Notably, as the thickness of DNSC increased, the clarity gradually decreased, resulting in visual discrepancies in the covered content (Figure S2). Therefore, for medical devices requiring high light transmittance (such as endoscopes), an appropriate thickness of DNSC is essential.

Wetting behavior and “slippery” performance are critical parameters for evaluating surface anti‐fouling strategies. To comprehensively assess its macroscopic anti‐adhesion effects, the contact angle (CA), sliding angle (SA), and contact hysteresis angle (CAH) were measured using various aqueous liquids, including deionized water (DI water), bacterial solution (with bacterial concentration of 1 × 10^6^–10^7^ CFU/mL), Dulbecco's Modified Eagle Medium containing 10% fetal bovine serum (DMEM+10% FBS) and blood. PG, LIS, and SLIPS were used as control groups. As shown in Figure 2G, compared with the PG surface, the DNSC‐COOH surface exhibited hydrophobic properties comparable to those of LIS and SLIPS, with a significant increase in CA (DI water from 42.6° to 111.6°, bacterial solution from 38° to 115.6°, DMEM+10%FBS from 54.6° to 105°, and blood from 53.7° to 107.7°). This hydrophobicity is likely attributed to the hydrophobic silicone oil framework at the coating's surface [36, 37]. Additionally, similar to LIS and SLIPS (Figure 2H), DNSC‐COOH exhibited significantly reduced SA and CAH for the droplets. The Rhodamine B‐labeled droplets (1 µg/mL) also demonstrated excellent adhesion to the modified surface, showing readily slippage (30° tilt, < 3 s) with no residue retention (Figure S3), whereas none of the liquids could slide off the PG surfaces under the same conditions (Figure 2I). These results indicated that DNSC‐COOH has excellent “slippery” characteristics, which will have a positive impact on the inhibition of biocontamination. To validate the broad applicability of DNSC‐COOH on surfaces of common non‐implantable/implantable devices, metal materials such as aluminum alloy (Al) and copper, along with polymer materials including silicon (Si), polyisoprene (Rubber), and polyethylene terephthalate (PET) were modified using the same method. The CA and SA of DI water on each surface were measured before and after modification. In all cases, the coated surfaces showed similar hydrophobicity and “slippery” performance (Figure S4), indicating that the superior “slippery” properties of the DNSC‐COOH modified surface originate from the coating's inherent “slippery” characteristics rather than being dependent on the substrate.

Furthermore, in order to better understand the inherent “slippery” characteristics of DNSC, the adhesion work of different liquids on the modified surface was calculated, because reducing the adhesion between the material and the substrate surface requires reducing the adhesion work. Specifically, based on the Owens‐Wendt‐Rabel‐Kaelble (OWRK) (ISO 19403‐2‐2017), and in combination with the Young equation and Van Oss equation, the adhesion work (W_a_) can be expressed as [38]:

$$W_{a}=\gamma_{l}\left(1+\cos\theta \right)$$

Among them, W_a_ means adhesion work, θ means the contact angle for the liquids. γ_l_ is the surface tension of the liquids, which can be measured by a contact angle measuring instrument. The specific measurement values are as follows: γ_l_ = ∼72.8 mJ/m for DI water, γ_l_ = ∼40 mJ/m for bacterial solution, γ_l_ = ∼ 52.1 mJ/m for DEME + 10% FBS, and γ_l_ = ∼60 mJ/m for blood. As shown in Figure 2J, compared with the PG surface, DNSC‐COOH had significantly reduced adhesion work to various liquids, thus showing excellent sliding characteristics, which will provide an important theoretical basis for effectively preventing bioadhesion.

### 2.3 Mechanical Wear Resistance and its Strengthening Mechanism of the Dendritic Nano‐Based Slippery Coating

**FIGURE 3:**
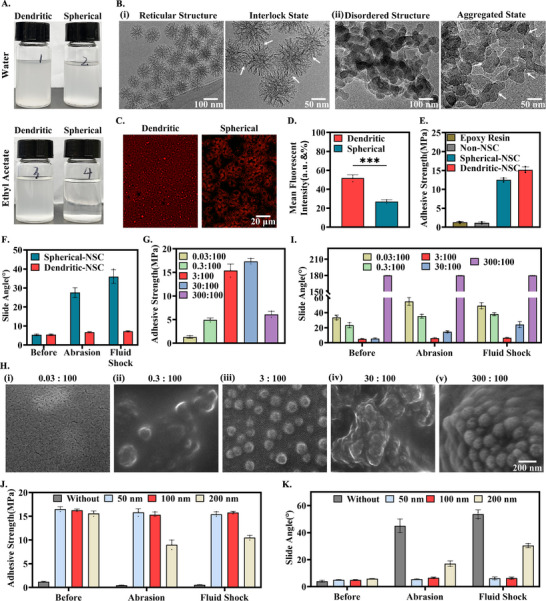
Mechanical wear resistance and its strengthening mechanism of the dendritic nano‐based slippery coating. (A) The aqueous phase dispersion system and the ethyl acetate dispersion system were formed by amino dendritic SiO_2_ nanoparticles and amino spherical SiO_2_ nanoparticles, respectively. (B) The TEM images showed that (i) the dendritic nanoparticles were uniformly dispersed and orderly reticular arranged and formed a physically interlocked state. On the contrary, (ii) spherical nanoparticles showed an obvious aggregated phenomenon. (C) Corn oil (a lubricant substitute) was fluorescently labeled with Nile red, and the particle distribution of the two morphologies and the amount of silicone oil adsorbed on the surface were observed under a fluorescence microscope. (D) Data statistics of fluorescence quantitative analysis of silicone oil adsorbed by nanoparticles of two morphologies. (E) Four types of NSC were respectively modified on the glass substrates, and the adhesion strength between the NSC and the glass was measured through the tape peeling test and statistically analyzed. (F) Similarly, the changes in the SA of the NSC constructed by the above two types of particles before and after sandpaper abrasion treatment and impact with the blood simulation agent. (G) Statistics on the adhesion strength of DNSC‐COOH with different ratios to glass substrates. (H) SEM images of the internal cross‐sections of DNSC‐COOH constructed with different mass ratios of dendritic nanoparticles and silicone oil. (I) The changes in the SA were observed when DNSC‐COOH with different ratios was subjected to sandpaper abrasion treatment and blood simulation agent impact treatment. (J) The adhesion strength and (K) SA changes of DNSC‐COOH with nanoparticles of different particle sizes before and after sandpaper abrasion treatment and impact with the blood simulation agent. Error bar represents the mean ± SD. *n* = 3, averaged.

The influence of nanoparticles — including their morphological effect, concentration, and size — on the mechanical properties of composite coating was systematically investigated, along with their strengthening mechanism. First, the effect of nanoparticle morphology on the mechanical properties of the coating was examined. A certain mass of amino dendritic nanoparticles and amino spherical nanoparticles were dispersed in aqueous and ethyl acetate solutions, respectively, to form suspensions (15 g/100 mL). As shown in Figure 3A, after standing for 6 h, the amino dendritic nanoparticles showed excellent dispersion in both aqueous and ethyl acetate solutions. In contrast, for the amino spherical nanoparticles, severe aggregation in ethyl acetate, and even when dispersed in aqueous, it was still inferior to that of dendritic nanoparticles. A transmission electron microscope (TEM) was used to further characterize the microscopic morphology of different particles in ethyl acetate. The results showed that, in stark contrast to the complete aggregation of spherical particles, dendritic particles not only remained uniformly dispersed, but also exhibited mechanical interlocking among their nanospikes, which would enhance the mechanical stability of the coating (Figure 3B). Subsequently, corn oil was used as a substitute for silicone oil and stained with Nile red. The impact of adsorbed oil on particle morphology was observed through fluorescence microscopy. As shown in Figure 3C, the spherical particle group exhibited numerous red aggregates, indicating agglomeration of spherical oil‐storing microparticles. In stark contrast, the dendritic particle group displayed uniform red distribution, demonstrating that dendritic particles uniformly captured the fluorescent oil layer while maintaining excellent dispersion. Notably, quantitative analysis of fluorescence intensity revealed significantly stronger red fluorescence on dendritic particles compared to ordinary spherical particles (Figure 3D). This suggests that dendritic nanoparticles, benefiting from their excellent dispersion, can capture more silicone oil, thereby ensuring the high “slippery” properties of DNSC. Furthermore, the mechanism by which dendritic nanostructures affect particle dispersibility was investigated. According to the Derjaguin‐Landau‐Verwey‐Overbeek (E_DLVO) theory, the total interaction potential between dendritic nanoparticles (V_E_DLVO_) is calculated as [39]:

VE_DLVO=VvdW+VDL+VHB

where V_vdW_, V_DL_, and V_HB_ represent van der Waals interactions, electrical double layer interactions, and hydrophobic interactions, respectively. Compared with ordinary spherical particles, the contact area between dendritic particles was reduced sharply due to the existence of nanospikes, resulting in a great decrease in the V_vdW_ and V_HB_, so that the amino dendritic nanoparticles can be dispersed without aggregation in ethyl acetate. For oil‐storing nanoparticles, the total interaction potential (V_E_DLVO_) between oil capture particles is:

VE_DLVO=VvdW+VDL+VLL

where V_LL_ represents lubricant‐lubricant interactions, respectively. Similarly, the nanospikes resulted in a decrease in the contact area between different oil‐capturing particles and a significant decrease in V_vdw_ and V_LL_. The reduction of attractive interactions between the particles allows the oil‐storing particles remain well dispersed in ethyl acetate without agglomeration.

Subsequently, the effects and strengthening mechanisms of the spike structure on the mechanical wear stability and slip performance of the coating, including both interfacial and bulk phases, were further revealed by adhesion strength and SA tests. The epoxy resin, nano‐based slippery coating (NSC) without nanoparticles (non‐NSC), NSC constructed with amino spherical particles (spherical‐NSC), and NSC constructed with amino dendritic particles (dendritic‐NSC) were coated on the glass surface via spin coating, and the adhesion strength of each coating was evaluated by tape peeling experiment. As shown in Figure 3E, the adhesion strength of epoxy resin and non‐NSC groups was very low (< 1.3 mPa). Although the adhesion strength of spherical‐NSC group was enhanced, the adhesion strength of the dendritic‐NSC group demonstrated significantly superior adhesive strength (> 15 mPa). Furthermore, two groups of samples, spherical‐NSC and dendritic‐NSC, were subjected to 500 cycles of sandpaper wear under a load of 50 grams, or dynamic artificial blood flow [40] (1750 s^−1^, arterial blood maximum shear rate, 15 days), respectively. The mechanical stability of dendritic‐NSC was further evaluated by the changes in SA. As shown in Figure 3F, the spherical‐NSC exhibited significantly increased SA, whereas the dendritic‐NSC maintains nearly unchanged, preserving its exceptional “slippery” performance. This enhancement is likely attributed to the outstanding dispersion stability of dendritic nanoparticles, which form a mechanically interlocked network within the epoxy matrix. This significantly improves coating stability and endows dendritic‐NSC with highly stable sliding properties.

Second, the influence of dendritic particle concentration on the mechanical properties of the coating was investigated. Different coatings were prepared by adjusting the mass ratio of particles to silicone oil in the DNSC‐COOH system (the mass ratio of particles to silicone oil was 0.03:100, 0.3:100, 3:100, 30:100, and 300:100, respectively). Similarly, the adhesion strength and mechanical stability of coatings were evaluated through tape‐peeling experiments and SA measurements after different treatment conditions. As shown in Figure 3G, DNSC‐COOH exhibited uniquely high adhesion strength (> 15 mPa) and mechanical stability when the particle‐to‐silicone oil mass ratio ranged from 3:100 to 30:100. Further scanning electron microscope (SEM) analysis revealed that as particle concentration increased (Figure 3H), uniformly dispersed nanoparticles gradually formed a dense dispersion‐continuum structure. However, at excessively high particle concentrations (m_particle_: m_silicone oil_ = 30/300:100), nanoparticle distribution became disordered, leading to reduced coating stability. Notably, the minimum DI water SA was observed at m_particle_: m_silicone oil_ = 3: 100 (Figure 3I). This may be attributed to the face that limited captured silicone oil at lower particle concentrations (0.03:100, 0.3:100), which negatively affects slip performance. Conversely, excessive particle concentration causes loose nanoparticle distribution, preventing water droplet wettability (Figure S5) and resulting in non‐sliding behavior. These results demonstrated that an appropriate nanoparticle ratio will significantly influence structural stability and “slippery” advantages in DNSC‐COOH systems. Therefore, considering both mechanical stability and slip performance, we selected the 3:100 particle‐to‐silicone oil mass ratio for subsequent studies.

Finally, the study investigated the effects of dendritic particle size on coating mechanical properties. By adjusting the size of amino dendritic nanoparticles (50, 100, and 200 nm) to prepare different DNSC‐COOH surfaces, the non‐NSC was used as the blank control group. Adhesion strength and mechanical stability were evaluated through tape‐peeling tests and SA measurements after various conditions. As shown in Figure 3J, nanoscale‐structured DNSC‐COOH surfaces exhibited increased adhesion energy compared to non‐NSC. However, the 200 nm group showed reduced adhesion energy compared to both the 50 and 100 nm groups. Similarly, after mechanical friction and fluid impact, the 50 and 100 nm groups maintained stable sliding behavior, while the 200 nm group demonstrated decreased sliding performance (Figure 3K). These results indicated that cross‐linked dendritic particles form a rigid, dense structure that enhances mechanical stability. Larger particle sizes lead to reduced interfacial adhesion energy between the composite coating and substrate, thereby compromising stability. This further emphasized that nanoparticle dispersion is a critical factor determining coating stability. The superior performance of dendritic nanoparticles stems from their anti‐agglomeration characteristics, which contrast sharply with spherical particles prone to forming larger aggregates. In conclusion, the maintenance of excellent mechanical sliding performance of DNSC‐COOH is attributed to the mechanical interlocking interaction between highly dispersed nanoparticles and nanospikes, appropriate concentration of particles, and the suitable particle size.

To further investigate whether the robust stability of DNSC‐COOH stems from molecular interactions between amino functionalized nanoparticles and silicone oils, control group coatings were prepared using silicone oils with ─OH and ─OCH_3_ terminal groups of identical viscosity (DNSC‐OH and DNSC‐OCH_3_). This selection establishes a clear comparative system based on the primary molecular forces involved: DNSC‐COOH relies on strong electrostatic interactions, DNSC‐OH on hydrogen bonding, and DNSC‐OCH_3_ on weaker van der Waals forces. This gradient of interaction intensity is designed to highlight the advantages of electrostatic interaction on the stability of the coating. Two experimental series were conducted: First, DNSC‐COOH and DNSC‐OH were immersed in 1 m NaCl, CaCl_2_, and FeCl_3_ solutions (1750 s^−^^1^, for 6 and 24 h). Second, neutral high‐speed blood flow impacts (1750 s^−^^1^, for 7 and 15 days) were applied to all three coatings. Stability was evaluated through measurements of DI water CA, SA, and CAH. Thickness and morphological changes after exposure to various stress conditions were also analyzed to determine whether swelling effects influence coating stability.

**FIGURE 4:**
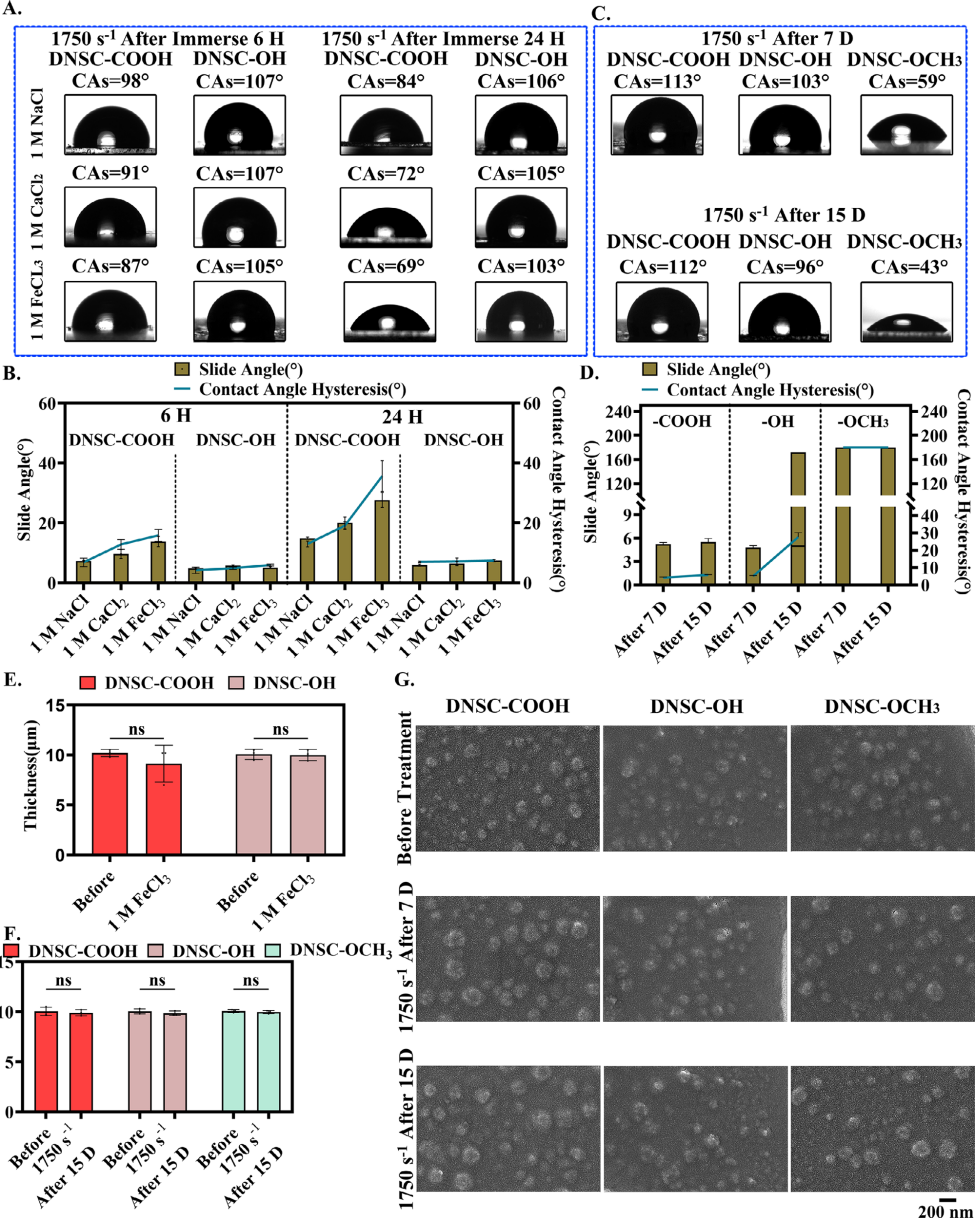
Mechanical wear resistance and its strengthening mechanism of the dendritic nano‐based slippery coating. (A,B) Three surfaces, DNSC‐COOH and DNSC‐OH were immersed in different ionic solutions (1750 s^−^^1^) to observe the changes in their hydrophobic properties and slip properties. (C,D) Three surfaces, DNSC‐COOH, DNSC‐OH, and DNSC‐OCH_3_, observed the changes in their hydrophobic properties and slip properties after being impacted by neutral high‐speed blood flow simulator for 7 and 15 days. (E,F) The thickness and (G) morphological changes comparison of the three surfaces before and after impact treatment with neutral high‐speed flow simulation agent for 7 and 15 days. Error bar represents the mean ± SD. *n* = 3, averaged.

As shown in Figure 4A,B, the hydrophobicity and slip performance of DNSC‐COOH significantly decreased under conditions of increased ionic strength and extended treatment duration, forming a stark contrast to the stable performance of DNSC‐OH. The results indicated that the carboxyl functional group may be damaged by electrostatic action due to the charge shielding effect in high ion concentration environments, and its stabilization mechanism fails [34, 41]. Notably, the excellent mechanical durability of DNSC‐COOH under neutral high‐speed blood flow was further corroborated. As shown in Figure S6, the surface elemental composition of the coating remained virtually unchanged even after 15 days of neutral high‐speed blood flow impact (1750 s^−^^1^). Also, it maintained basic stability in CA, SA, and CAH (Figure 4C,D), confirming the stable immobilization of the lubricant and the integrity of the composite matrix. In comparison, DNSC‐OCH_3_ nearly completely lost its slip behavior within 7 days, while DNSC‐OH exhibited significant performance degradation after 15 days. Although none of the coatings demonstrated swelling‐induced thickness increase (Figure 4E,F) or cross‐sectional morphological changes (e.g., cracks or swelling) post‐challenge (Figure 4G), their functional stability exhibited notable differences. These findings strongly suggested that molecular forces dominated by different chemical functional groups exhibit fundamental differences in responding to environmental challenges. On the one hand, DNSC‐COOH relies on electrostatic interactions, which provide exceptional stability in neutral fluids but are easily disrupted by metal ions in ionic solutions. On the other hand, DNSC‐OH may achieve certain stability through hydrogen bonding, though its strength is far less than that of electrostatic interactions (Figure S7). It is thus reasonable to speculate that the superior performance of DNSC‐COOH over DNSC‐OH under neutral conditions indicated that electrostatic interactions may play a more dominant role than hydrogen bonds in maintaining the dynamic stability of the coating. The stability of the coating is determined not only by the mechanical strength of the particles themselves, but also by electrostatic interactions with surrounding media (such as silicone oil). These two factors collectively determine the coating's durability and functionality in complex environments.

### 2.4 Antibiological Substance Adhesion and Accumulation Effects of the Dendritic Nano‐Based Slippery Coating

When medical devices such as catheters come into contact with blood, the adsorption of physiological proteins activates biological processes including the coagulation cascade. The triggers platelet activation, cell adhesion, and aggregation, ultimately leading to device‐associated inflammation and thrombosis that significantly impair clinical performance [42, 43]. To systematically evaluate the anti‐biofouling properties of DNSC‐MC, this study investigates their adhesion behavior on modified surfaces from three perspectives: protein, bacterial, and cellular engagement.

**FIGURE 5:**
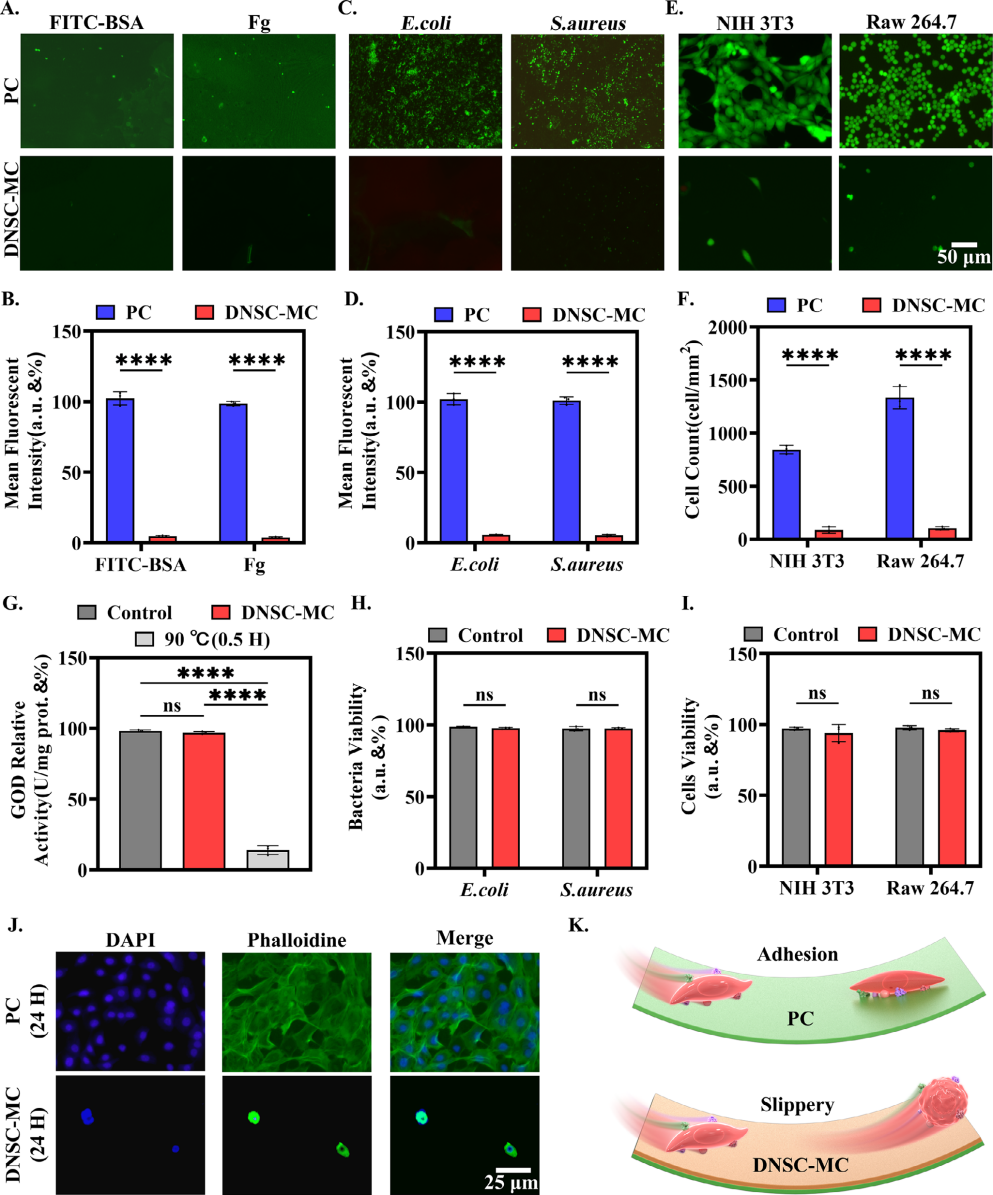
Antibiological substance adhesion and accumulation effects of the dendritic nano‐based slippery coating. (A,B) Fluorescence representative images of adhesion of two proteins (FITC‐BSA, Fg) on two surfaces and quantitative analysis statistics of fluorescence intensity. (C,D) Fluorescence representative images of adhesion of two types of bacteria (*E. coli, S. aureus*) on two surfaces and quantitative analysis statistics of fluorescence intensity. (E,F) Fluorescence representation images and cell count statistics of adhesion of two types of cells (NIH 3T3, Raw 264.7) on two surfaces. Summary of the research results on the activity of DNSC‐COOH in (G) proteins, (H) bacteria and (I) cells. (J,K) The interaction and mechanism diagram of the cytoskeleton of NIH 3T3 cells on two surfaces were observed through surface morphology. Error bar represents the mean ± SD. *n* = 3, averaged.

First, using fluorescein isothiocyanate‐labeled bovine serum albumin (FITC‐BSA, 5.12 mg/mL) and fluorescent fibrinogen (Fg, 5 mg/mL) were used as model proteins to evaluate the resistance of DNSC‐MC surface to protein adsorption, with the PC surface serving as the control. The surfaces were statically incubated with protein solutions at 37°C for 24 h, respectively. Fluorescence analysis was performed post‐incubation to quantify protein adsorption levels. Representative fluorescence images in Figure 5A showed significant protein adsorption on PC surfaces compared to DNSC‐MC surfaces. Quantitative fluorescence intensity analysis (Figure 5B) further demonstrated that protein adsorption on DNSC‐MC surfaces dropped below 5%, representing at least 21 times reduction compared to PC surfaces. Which indicated that the DNSC‐COOH coating effectively inhibits protein adsorption and blocks the initial stage of thrombus formation.

To investigate the inhibitory effects of the surface on bacterial biofilms, two common pathogens — *Escherichia coli (E. coli*) and *Staphylococcus aureus* (*S. aureus*) were tested through adhesion experiments. After 24 h static co‐cultivation at 37°C, the bacteria were fluorescently labeled and observed under a fluorescence microscope. The results (Figure 5C) showed minimal green fluorescence on the DNSC‐MC surface, indicating low bacterial adhesion. In contrast, the PC surface was densely covered with both *E. coli* and *S. aureus*. Quantitative analysis (Figure 5D) revealed that bacterial adsorption on DNSC‐MC approached 5.4%, which was at least 18 times lower than on PC. This demonstrated that the DNSC‐COOH surface exhibits excellent anti‐bacterial adhesion resistance, effectively preventing biofilm formation and potentially reducing medical device‐associated infection risks at the source.

Furthermore, hematogenous immune cells (such as macrophages) can induce inflammation and thrombosis through recognition and adhesion to foreign surfaces. The inhibitory effect of DNSC‐MC on cellular adhesion was further evaluated by using mouse embryonic fibroblasts (NIH 3T3, which were kindly provided by Wuhan Procell) and mononuclear macrophages (Raw 264.7, were kindly provided by Wuhan Procell) as models. DNSC‐MC samples were co‐incubated with cells at 37°C for 24 h and then observed their adhesion to the material surface using Calcein‐AM fluorescence staining. PC was used as a control. As shown in Figure 5E,F, PC exhibited dense cell coverage on its surface, while DNSC‐MC only showed sporadic green fluorescent cell adhesion. Quantitative analysis revealed that the cell density per unit area on DNSC‐MC surfaces (NIH 3T3: 87 cells/mm^2^, Raw 264.7: 103 cells/mm^2^) decreased by over 9.7 times compared to the PC group, demonstrating superior anti‐adhesion performance.

To elucidate the mechanism of DNSC‐MC's anti‐bioadhesion properties, multidimensional experiments were conducted to systematically evaluate its potential biological toxicity effects, combined with surface physical characteristics and cellular morphology analysis. First, the impact of DNSC‐MC on proteins, bacteria, and cell viability was comprehensively assessed. At the molecular level, glucose oxidase (GOD) was used as a model protein. DNSC‐MC was co‐incubated with GOD solution at 37°C for 24 h, and enzyme activity changes were measured using a GOD assay kit. Untreated PC and heat‐denatured (boiling water bath treatment) samples served as control groups. The results (Figure 5G) showed no significant difference in GOD activity between the DNSC‐MC treatment group and the blank control, while the heat‐denatured group exhibited nearly complete loss of enzyme activity. This indicated that DNSC‐MC does not achieve antifouling effects by disrupting protein structure or function. At the bacterial level, co‐culturing DNSC‐MC with *E. coli* or *S. aureus* at 37°C for 24 h was performed, with medium without DNSC‐MC serving as the negative control. Fluorescence detection was performed with live/dead bacterial staining reagent. As shown in Figure S8, no significant dead bacteria fluorescence signal (red) was detected in the two groups. Statistics (Figure 5H) showed that the bacterial survival rate in the DNSC‐MC group was greater than 97%, and there was no statistical difference with the control group (p > 0.05), which proved that DNSC‐MC did not effect on bacterial vitality. To further evaluate its biocompatibility at the cellular level, NIH 3T3 and Raw 264.7 cells were selected to be co‐cultured with DNSC‐MC under the same conditions, and the cytotoxicity was evaluated by using a live/dead staining reagent. Fluorescence images (Figure S8) revealed no detectable PI red fluorescent cells in either the experimental or control group. Statistical analysis (Figure 5I) demonstrated that both cell lines maintained survival rates exceeding 93% under DNSC‐MC exposure, showing no significant difference compared to the control group. This confirmed that DNSC‐MC exhibits no apparent cytotoxicity toward mammalian cells and the DNSC‐MC's anti‐biofouling properties are not mediated through toxic mechanisms such as inactivating biomolecules, killing microorganisms, or disrupting cellular functions.

In order to further explore its anti‐adhesion mechanism, the surface characteristics and cell behavior were analyzed in depth. Theoretical analysis showed that DNSC‐MC has very low interfacial energy and unique slippery characteristics, which can make a variety of water‐based liquids including protein solution, cell culture medium, and whole blood slide quickly without residue. This property makes it difficult for biomolecules to adsorb effectively on the surface through forces such as hydrophobic interactions, ionic bonds or hydrogen bonds. In a dynamic fluid environment, protein molecules are easily carried away by the shear force of the fluid. Even under static conditions, they tend to diffuse into the bulk solution due to the lack of effective binding sites. Adhesion of bacteria and cells to a surface is usually dependent on adhesins (such as bacterial cilia, flagella) or integrin‐ligand interactions in eukaryotic cells. The continuous sliding characteristics of the DNSC‐MC surface and the lack of a protein preadsorption layer prevent the anchoring of these specific adhesion structures, thus fundamentally inhibiting bioadhesion. In order to verify this mechanism from the perspective of morphology, NIH 3T3 cell was used as a model. After co‐culturing with DNSC‐MC for 24 h, nuclei were stained with 4',6‐diamidino‐2‐phenylindole (DAPI) and actin filaments were labeled with phalloidine. The cell spreading morphology was observed by fluorescence microscope. Meanwhile, an unmodified PC was used as the control group. As shown in Figure 5J,K, the PC surface cells were widely spread and the skeleton was well developed, which may benefit from the higher protein adsorption to provide more adhesion sites and thus exhibit strong adhesion. In sharp contrast, the surface cells of DNSC‐MC were round and had a small spreading area, which further indicated that the surface of DNSC‐MC effectively inhibits cell adhesion and spreading. Therefore, it is proven again that DNSC‐MC has unique surface physical smooth characteristics — rather than bio‐toxic effects — to achieve highly effective anti‐biofouling properties. The material's surface energy significantly inhibits protein adsorption, bacterial adhesion, and cell spreading, demonstrating excellent biocompatibility and anti‐adhesion performance. This makes it a promising candidate for biomedical applications such as implantable medical devices and in vitro diagnostic equipment, with the potential to substantially reduce risks of device‐related infections and thrombosis.

### 2.5 Antithrombotic Effect and In Vivo Biosafety of the Dendritic Nano‐Based Slippery Coating

**FIGURE 6:**
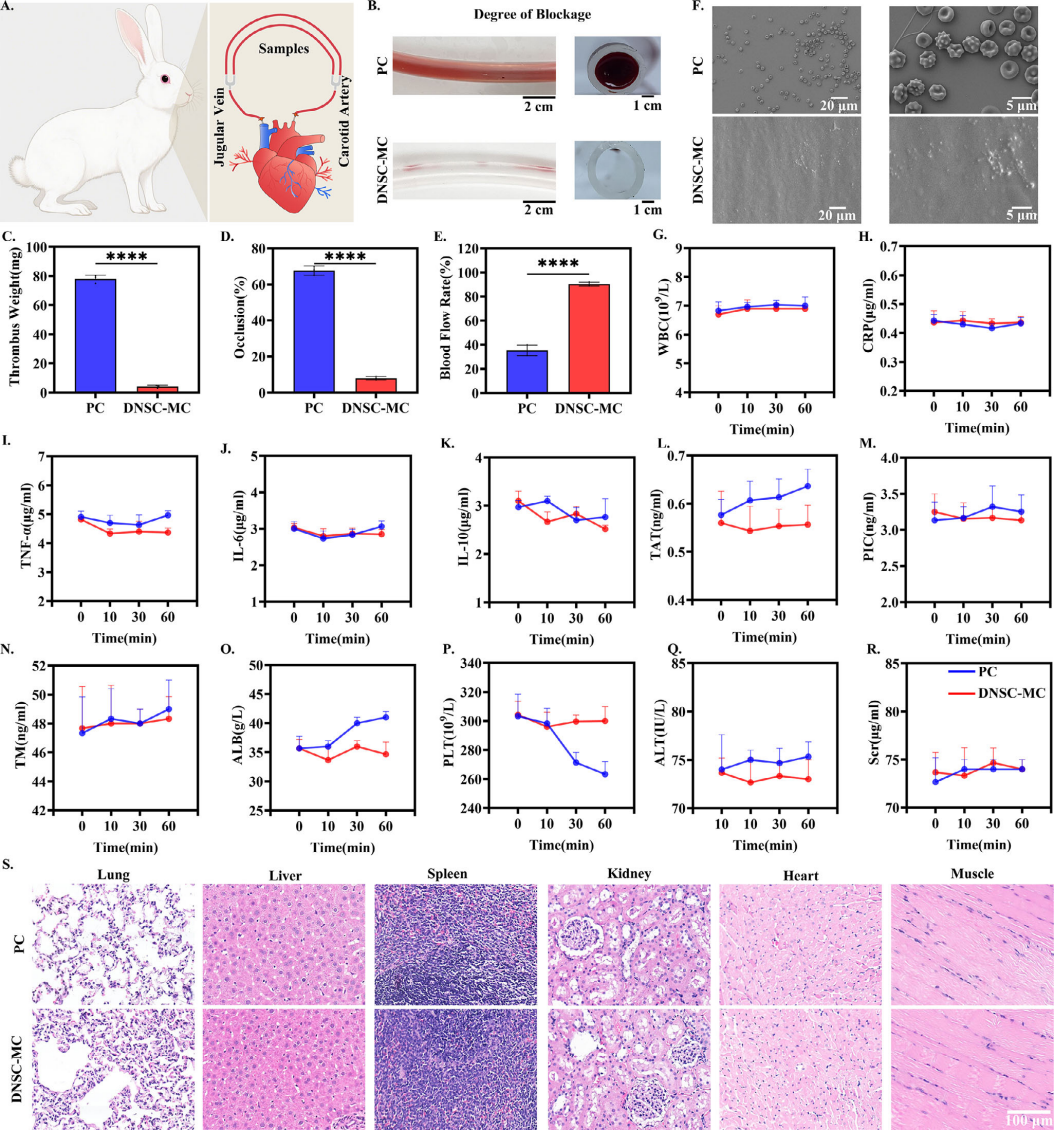
Antithrombotic effect and in vivo biosafety of the dendritic nano‐based slippery coating. (A) Schematic diagram of the in vitro circulation model of the closed‐loop catheter in New Zealand rabbit. (B) The coagulation and occlusion conditions in the lumen of the two groups of catheters after 2 h of circulation. (C–E) The total weight of thrombi formed in the lumen of the two groups of catheter samples after 2 h of circulation, the lumen occlusion degree and blood flow rate statistics tables. (F) SEM images showed the platelet adhesion on the lumen surfaces of the two groups of catheters in the extracorporeal whole blood closed circulation system after 2 h. After 1 h in the arteriovenous shunt circuit of New Zealand rabbits, blood was collected at different time points (0, 10, 30, 60 min) for the comparison of (G–K) inflammatory factor contents, (L–P) coagulation indicators, (Q) Indicators of liver and (R) kidney function impairment. (S) H&E staining results of inflammatory responses in various organs and muscle tissues at the implantation sited 15 days after PC and DNSC‐MC samples were implanted into the subcutaneous tissues of rats. Error bar represents the mean ± SD. *n* = 3, averaged.

Thrombosis represents a core challenge for blood contact implantable devices, primarily driven by surface‐induced adsorption of fibrinogen and subsequent platelet adhesion and activation [43]. Based on the excellent broad‐spectrum anti‐biofilm adhesion properties of DNSC‐MC, its anti‐thrombosis performance was further investigated. Commercial sterile PVC catheter, DNSC‐MC and commercial central venous catheter were assembled and connected to the arteriovenous shunt circuit of rabbits to establish a closed‐loop catheter circulatory model in vitro in rabbits (Figure 6A). After 2 h of in vitro circulation without any anticoagulant, all catheters were collected to observe thrombosis formation. Thrombus weight, catheter occlusion rate and blood flow velocity were measured to comprehensively evaluate its antithrombotic performance. As shown in Figure 6B, the catheter wall of the PC group was seen to have a large number of blood clots, while the DNSC‐MC group showed excellent anticoagulant effect with transparent catheter wall and no obvious thrombosis. Quantitative analysis further confirmed this observation, compared to PC, as evidenced by the significant reduction of DNSC‐MC in thrombus weight (Figure 6C, 78 vs. 4.1 mg), occlusion rate ((Figure 6D, 67.6% vs. 8%), and blood flow rate (Figure 6E, 35.3% vs. 90.3%).

In order to further clarify the mechanism of DNSC‐MC inhibition of thrombosis, an in vitro whole blood closed circulation system was constructed, and DNSC‐MC and PC materials were connected in series into the circuit, and fresh whole blood labeled with Fg was used for circulation experiments. After 2 h of circulation, the adhesion of fibrinogen on the surface of the catheter was quantitatively observed by fluorescence microscope, and the platelet adhesion morphology on the surface was analyzed by SEM. As shown in Figure S9, the inner surface of the PC lumen exhibited substantial fibrinogen adsorption, resulting in significant fluorescence accumulation. In contrast, the DNSC‐MC surface displayed only sporadic fluorescent signals. This demonstrated that the DNSC‐MC can effectively inhibit fibrinogen adhesion even in dynamic fluid environments, thereby preventing its activation and subsequent triggering of the coagulation cascade. The SEM results further supported the aforementioned conclusions: The PC surface exhibited numerous platelet aggregates and activated forms, while the DNSC‐MC surface only showed scattered blood cell adhesions with no significant platelet adhesion or activation structures. Therefore, DNSC‐MC fundamentally inhibits platelet adhesion and activation by significantly reducing fibrinogen initial adsorption, thereby effectively preventing medical device‐related thrombus formation.

For implantable biomedical devices such as central venous catheters, in addition to anti‐thrombotic performance, the safety of their direct contact with blood in vivo is also crucial, and the effects on blood components, immune function, liver and kidney, and other important organs need to be comprehensively evaluated. In this study, DNSC‐MC was placed in the arteriovenous shunt circuit of rabbits, and blood samples were collected at different time intervals (0, 10, 30, and 60 min), and a number of physiological and biochemical indexes including inflammation, coagulation and organ function were systematically analyzed. In terms of inflammation, there was no significant change in pro‐inflammatory parameters in all groups (Figure 6G–K), including white blood cell (WBC), C‐reactive protein (CPR), tumor necrosis factor‐α (TNF‐α), and inflammatory and immunosuppressive representative factors (IL‐6, IL‐10). Compared with the starting point (0 min), both the PC group and the DNSC‐MC group fluctuated within the normal range, indicating that DNSC‐MC did not further promote the material inflammatory response. Regarding coagulation function, measurements of key biomarkers including thrombin‐antithrombin complex (TAT), plasmin‐α2 antithrombin complex (PIC), and thromboxane receptor (TM) revealed that all parameters in the DNSC‐MC group remained within normal fluctuation ranges. In contrast, the PC group exhibited an upward trend, indicating enhanced activation of the coagulation pathway following blood exposure (Figure 6L–O). In addition, the platelet (PLT) count in the PC group decreased over time, which may be attributed to platelet adhesion and consumption on the surface of the material. In contrast, the PLT level in the DNSC‐MC group remained basically stable, further confirming its good anticoagulant performance (Figure 6P). To further evaluate the impact of the material on organ function, alanine aminotransferase (ALT) reflecting liver function and serum creatinine (Scr) indicating kidney function were measured. As shown in Figure 6Q,R, both ALT and Scr levels remained stable during circulation in both groups, demonstrating that DNSC‐MC exhibits no toxic effects on hepatic or renal functions like PC. This finding further confirmed its excellent biosafety and hemocompatibility.

Furthermore, hemolytic reaction assessment of biomaterials serves as a critical indicator for translating biomedical applications. Fresh rabbit blood was collected for in vitro hemolysis experiments to evaluate the in vitro blood compatibility of DNSC‐MC. As shown in Figure S10 the DNSC‐MC exhibited no hemolysis with a hemolysis rate as low as 3.1%, meeting the clinical application standard (< 5%) [44]. Considering the detachment of coating nanoparticles and other potential biological toxicities, the biosafety of DNSC‐COOH in vivo was further evaluated. The DNSC‐MC was subcutaneously implanted for 15 days, and the inflammatory changes and foreign body reactions in various organs (lungs, liver, spleen, kidney, and heart) and muscle tissues at the implantation site were observed by hematoxylin and eosin (H&E) staining. Meanwhile, the unmodified PC was used as the control group. As shown in Figure 6S, no abnormal inflammatory cell infiltration was observed in the aforementioned organs and implantation sites, with normal cell counts and morphology. The results indicated that regardless of whether the particles were released or not, DNSC‐MC demonstrates excellent in vivo biocompatibility by not inducing inflammation or foreign body reactions in organs and tissues under physiological conditions.

### 2.6 Long‐Term Durability in Antibiological Adhesion and Anti‐Thrombosis of the Dendritic Nano‐Based Slippery Coating

**FIGURE 7:**
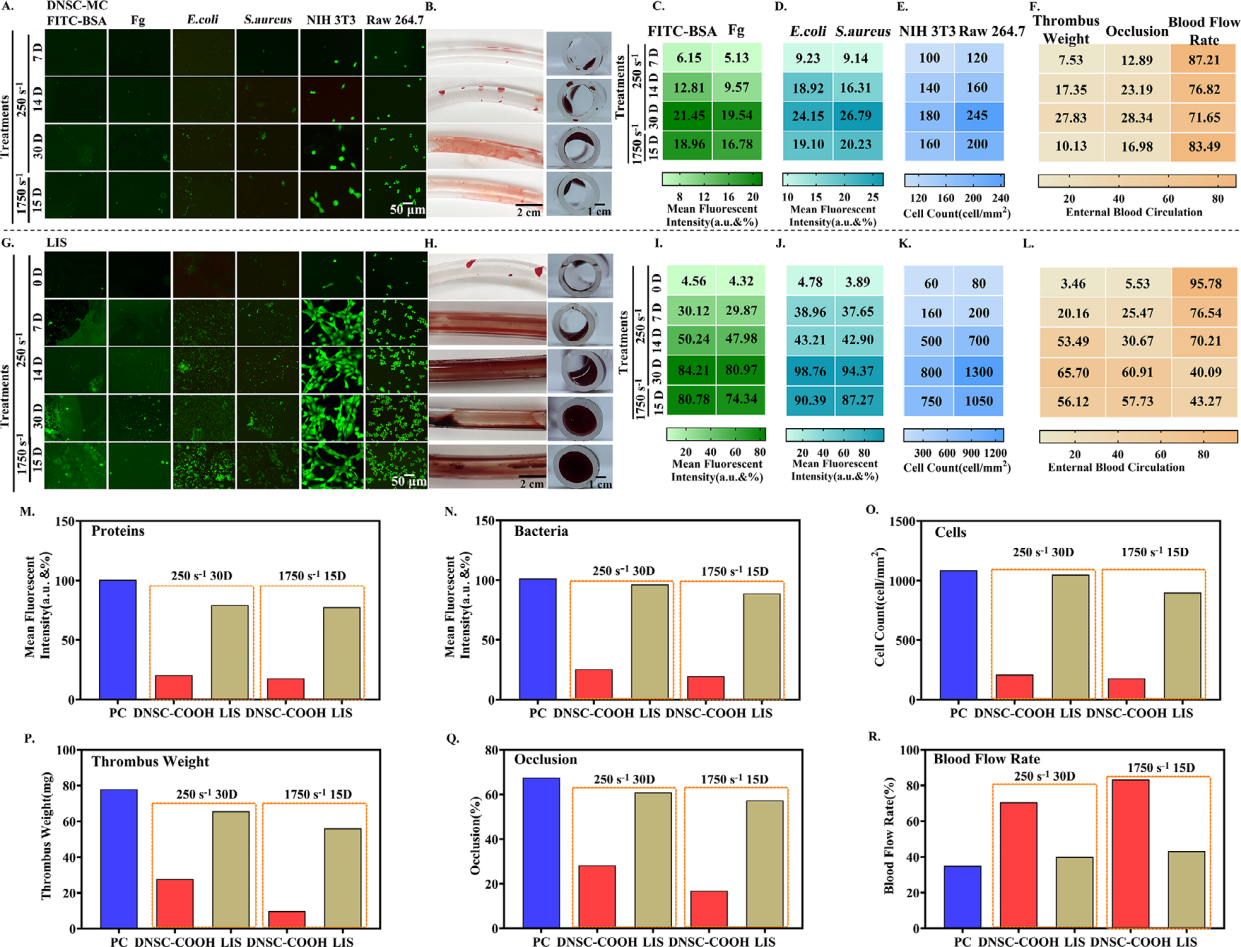
Long‐term advantages in anti‐biological adhesion and anti‐thrombosis of the dendritic nano‐based slippery coating. (A) Fluorescence representative images of proteins, bacteria, and cells anti‐adhesion experiments conducted on DNSC‐MC after fluid shock under different conditions. (B) The thrombosis and occlusion conditions inside the lumen of DNSC‐MC after being subjected to fluid shock under different conditions for 2 h. (C–E) Quantitative analysis statistics table of proteins, bacterial and cells anti‐adhesion experiments of DNSC‐MC under different conditions after fluid impact. (F) Statistics of total thrombus weight, catheter occlusion degree, and blood flow rate in the lumen of DNSC‐MC 2 h after fluid impact under different conditions. (G) Fluorescence representative images of proteins, bacteria, and cells anti‐adhesion experiments conducted on LIS modified cathers after fluid shock under different conditions. (H) The thrombosis and occlusion conditions inside the lumen of LIS modified catheters after being subjected to fluid impact under different conditions for 2 h. (I–K) Quantitative analysis statistics table of proteins, bacterial, and cells anti‐adhesion experiments of LIS modified catheters under different conditions after fluid impact. (L) Statistics of total thrombus weight, catheter occlusion degree, and blood flow rate in the lumen of LIS modified catheters 2 h after fluid impact under different conditions. (M–O) Quantitative analysis of proteins, bacteria and cells adhesion results of the DNSC‐MC, LIS modified catheters and PC groups after treatments at the shear rate of 250 s^−1^ for 30 days and 1750 s^−1^ for 15 days. (P–R) Quantitative analysis of total thrombus weight, catheter occlusion degree, and blood flow rate results of the three groups after above treatments. Error bar represents the mean ± SD. *n* = 3, averaged.

The long‐term durability of surface functions is crucial for indwelling biomedical devices. To simulate the real blood flow environment, the durability of DNSC‐COOH was evaluated by subjecting it to long‐term dynamic blood flow environment impacts [40] (with a blood simulant of viscosity approximately 4 mPa·s as the medium, at low shear rate of 250 s^−^^1^ for 7, 14, and 30 days, and at high shear rate of 1750 s^−^^1^ for 15 days). After dynamic fluid treatment, anti‐biofouling and blood circulation experiments in vitro were conducted. Catheters modified with LIS served as the control groups. As shown in Figure 7A–L, under long‐term blood environment challenges, regardless of the low shear rate of ∼ 250 s^−^^1^ for 30 days or the high shear rate of ∼ 1750 s^−^^1^ for 15 days, DNSC‐COOH still maintained excellent anti‐biofouling performance, with inhibition rates of proteins, bacteria, and cells remaining at least 80%, 76%, and 80.5%, respectively. In contrast, for LIS, although it maintained excellent anti‐adhesion performance for a certain period of time at low shear rate (< 7 days), its anti‐adhesion performance gradually decreased with increasing flow rate or time, which might be due to the loss of surface lubricants (Figure S11). Similarly, although the anti‐thrombotic efficiency of the DNSC‐COOH catheter blood circuit slightly decreased after long‐term dynamic blood flow environment impacts, it still maintained substantial effectiveness as assessed by thrombus weight, occlusion ratio, and blood flow rate, with more than 71.7% and 83.1% of its initial capacity preserved at ∼ 250 s^−^^1^ after 30 days and at ∼ 1750 s^−^^1^ after 15 days (Figure 7M–R). Conversely, LIS‐modified catheters exhibited extensive thrombus formation, retaining less than 42.7% of their initial capacity post‐flow exposure, indicating a significant decline in long‐term anti‐thrombogenic performance. These results indicated that DNSC‐COOH can stably resist the adhesion of proteins, bacteria, and cells in a dynamic blood flow environment for a long time and maintain excellent anti‐thrombotic performance, highlighting its significant potential in constructing anti‐thrombotic surfaces for long‐term implantable medical materials.

To evaluate the stability of DNSC‐MC in clinical environments, it was subjected to high pressure (0.1 mPa, 120°C, 30 min), ultraviolet irradiation (the ultraviolet intensity is approximately 70 µW/cm^2^, for 60 min), ultrasonic cleaning (20 kHz, for 30 min, UC‐9480, Ulangee), repeated friction with sandpaper simulating the vascular wall (1500# sandpaper, 1000 times), and immersion in physiological environment simulation solutions (pH 1, pH 5.5, pH 7.5, and 0.9% NaCl solution, for 30 days), and the changes in its sliding performance and anti‐protein adhesion performance before and after treatment were systematically evaluated. As shown in Figures S12–S16, after most environmental treatments, the sliding performance and anti‐protein adhesion performance of DNSC‐MC did not change significantly. However, after being immersed in the pH 1 solution for 30 days, the protein adhesion fluorescence intensity significantly increased from 4.87% to 57.5%, and the SA increased to 42.5°, indicating a severe decline in the surface anti‐adhesion and lubrication performance. This might be attributed to the strong acidic environment that disrupted the electrostatic interaction in the DNSC‐COOH system, resulting in a significant functional impairment [45]. Except for this extreme condition, DNSC‐MC maintained excellent performance after all other treatments, demonstrating its stability and suitability for application on various clinical devices/equipment surfaces.

## 3 Conclusion

In summary, the study presents a dendritic structure‐based slippery coating, fabricated by immobilizing ─COOH silicone oil via amino functionalized dendritic nanoparticles, and subsequently integrated into an epoxy resin matrix. The coating demonstrates significant improvement in the long‐term anti‐biofouling and antithrombotic performance of implantable blood‐contacting medical devices. The nanoparticles demonstrate high dispersion and enhance mechanical interlocking attributed to their dendritic topological structure, while electrostatic interactions between synergistic amino and carboxyl groups effectively immobilize the lubricating components. The synergistic effect substantially enhances both mechanical robustness and slipperiness. Under simulated blood shear conditions (shear force ∼ 1750 s^−^^1^), DNSC‐COOH demonstrated an unprecedented anti‐adhesion ability against proteins, bacteria, mammalian cells, and platelets for more than 15 days. Systematic characterization and biological evaluations confirmed that the anti‐adhesion performance originates from the coating's physical “slippery” characteristics rather than bioactive substance release, with excellent biocompatibility. Subsequent in vitro and in vivo experiments revealed that DNSC‐MC effectively inhibits thrombosis over extended periods without inducing significant inflammatory responses or tissue damage. Through coordinated optimization of structural design and interfacial anchoring, this study successfully addresses the challenge of balancing mechanical stability and surface anti‐adhesion properties in dynamic flow environments. Such slippery coating exhibits superior anti‐biofouling and durability under shear conditions like blood flow, providing a highly biocompatible surface solution for implantable devices operating in fluidic environments such as vascular catheters. We expect that the strategy will not only effectively inhibit thrombosis and biological contamination but also open up a new technical pathway for the development of long‐term implantable devices in complex and dynamic physiological environments. Future work will focus on introducing self‐healing mechanisms to further extend the coating's service life under extreme conditions.

## Statistical Analysis

Each experiment was performed in at least four replicates. Data are presented as the mean ± standard deviation (mean ± SD). Using the GraphPad Prism.9. 5 Software and Origin 2024 software was performed the statistical analysis. Statistical analysis between multiple groups used one‐way analysis of variance (ANOVA) followed by Tukey post hoc test and two‐sample t‐test for the comparison between two samples.

## Author Contributions

S.Z. and C.D.Y. designed experiments, analyzed data, and wrote the manuscript. S.Z., Y.S., J.L., Q.Z., T.Y.N., S.P.C., Y.Z.M., X.Y.Q., J.Y.Y., and T.F. performed experiments. G.Z.H., J.H.Z., and C.D.Y. provided conceptual advice and supervised the study. All authors discussed the results and assisted in the preparation of the manuscript.

## Funding

The authors would like to acknowledge the following funding which has provided the necessary financial support for this work. Basic and Applied Basic Research Foundation of Guangdong Province (Grant Nos. 2023A1515110332, 2025A1515010615, and 2022A1515012460), Basic and Applied Basic Research Foundation of Guangzhou (Grant No. 2024A04J3464), The construction of high‐level talents in high‐level disciplines in high‐level universities of Southern Medical University (22G601), National Natural Science Foundation of China (82072528), Guangdong Provincial Young Scientific and Technological Talent Cultivation Program (Grant No. SKXRC2025160).

## Conflicts of Interest

The authors declare no conflicts of interest.

## Supporting information




**Supporting File**: advs74123‐sup‐0001‐SuppMat.docx.

## Data Availability

The data that support the findings of this study are available in the supplementary material of this article.
