# Supplementary material for: Dendritic Nano‐Based Slippery Coating by Synergistic Mechanical and Electrostatic Interactions with Persistent and Exceptional Combats Thrombosis
Source: Adv Sci (Weinh). 2026 Jan 29;13(19):e21672. doi: 10.1002/advs.202521672 (PMC13045410; doi:10.1002/advs.202521672)
Supplement: Supplementary file 1 — Supporting File: advs74123‐sup‐0001‐SuppMat.docx. [file ADVS-13-e21672-s001.docx]

**Dendritic Nano-Based Slippery Coating by Synergistic Mechanical and Electrostatic Interactions with Persistent and Exceptional Combats Thrombosis**

Shu Zhang^1^, Yao Shen^1^, Juan Liu^2^, Qing Zeng^1^, Yunze Ma^1^, Shuping Chen^1^, Taiyu Nan^1^, Xiaoying Qiu^1^, Jiayi Yu^1^, Tao Fan^1^, Guozhi Huang^1^*, Jihua Zou^1^*, Chengduan Yang^1^*

^1^Center of Rehabilitation Medicine, Zhujiang Hospital, School of Rehabilitation Sciences, Southern Medical University, Guangzhou, China

^2^Rice Research Institute, Guang Dong Academy of Agricultural Sciences, Guangzhou, China

*To whom correspondence may be addressed. Corresponding to: Chengduan Yang, [yangchd13@smu.edu.cn;](mailto:yangchd13@smu.edu.cn;) Jihua Zou, [zoujihua@smu.edu.cn;](mailto:zengqingyang203@126.com;) Guozhi [Huang, drhuang66@163.com;](mailto:Huang,drhuang66@163.com;)

**Experimental Section**

**Materials**

Amino dendritic silica nanoparticles (customized by Xi'AN QIYUE BIOLOGY) (particle size 50 nm, 100 nm, 300 nm), aminated spherical silica nanoparticles (100 nm, Xi'AN QIYUE BIOLOGY), silicone oil (viscosity 10 cSt, including -COOH end (customized by Dongguan ZILIBON), -OH end (Sigma-Aldrich) and -OCH_3_ end (Sigma-Aldrich), ethyl acetate (analytical grade), and silane coupling agent (Shandong Yousuo Chemical Technology Co., LTD.). Trichloro-1H, 1H,2H, 2H-perfluorooctane silane (TFOS, Sigma-Aldrich), anhydrous ethanol, perfluorinated naphthalene (PFD, Sigma-Aldrich). Hydrofluoric acid (HF, concentration 50%, Sigma-Aldrich), saturated sodium bicarbonate solution (Aladdin), artificial blood (pH = 7.3, viscosity was about 4 mPa·s). Medical polyethylene (PVC) sterile catheter (inner diameter 3mm, outer diameter 4mm), glass (18mm × 18mm × 0.13mm, BKMAM), copper, aluminum (1060#, 35mm × 15mm × 2mm), silicon, Polyisoprene (rubber) and polyethylene terephthalate (PET).

**Preparation of the dendritic nano-based slippery coating**

At room temperature, the amino dendritic nanoparticles, silicone oil, and silane coupling agent were mixed in an initial mass ratio of 3:100:50 and stirred overnight to obtain solution A. Dissolve the epoxy resin in ethyl acetate to obtain solution B. The two solutions were thoroughly mixed and stirred for 12 hours to complete cross-linking and obtain a semi-transparent DNSC solution.

The morphology of nanoparticles (dendritic, spherical) was adjusted respectively, and two other types of NSC (dendritic-NSC, spherical-NSC) were obtained by the same preparation method. The mass ratio of dendritic nanoparticles to carboxyl (-COOH) silicone oil was adjusted (0.03:100, 0.3:100, 30:100, 300:100) to obtain another four types of DNSC-COOH. Two other types of DNSC-COOH were obtained by adjusting the particle size of dendritic nanoparticles (50 nm, 300 nm). Adjust the type of silicone oil (hydroxyl silicone oil, methoxy silicone oil) to obtain two other different DNSC (DNSC-OH, DNSC-OCH_3_).

**Plasma surface treatment**

Specific process parameters: PVC catheter, glass, copper, aluminum, silicon. The rubber and PET surfaces were all exposed to a vacuum environment of 80 pa at a frequency of 40 kHz for 3 minutes (O_2_, TS-PL05, Tonson Tech).

**Spraying and thickness measurement**

The spraying parameters are as follows: Atomize and spray for 3 seconds at a distance of 15cm (with a solution concentration of 100%) (with a thickness control of approximately 10 μm). The spraying time was extended to 6, 12 and 18 seconds, and DNSC-COOH with thicknesses of 20, 40 and 60 μm were obtained respectively.

After the sample spontaneously cured overnight on the substrate surface, the thickness of the DNSC-COOH was measured using a Japanese Sanliang CT400 paint film thickness gauge. Unless otherwise specified, DNSC-COOH with a thickness of 10 μm was selected as the research object. The subsequent thickness tests also adopt this approach for thickness measurement.

**Preparation of liquid-infused surfaces (LIS)**

The original glass and original PVC catheter samples were immersed in liquid silane solution (TFOS) at 80 °C for 12 hours. The treated samples were rinsed three times with anhydrous ethanol and deionized water. The cleaned samples were slowly heated overnight at 60 °C atmospheric pressure to obtain covalent grafted layers. Then, apply the movable liquid perfluorocarbon layer. The coated sample was impregnated in PFD. Finally, the LIS modified surface was obtained.

**Preparation of liquid-infused surfaces (SLIPS)**

Select the glass substrate for modification. First, clean glass needs to be etched with HF for 5 minutes. Then they were respectively immersed in deionized water and saturated sodium bicarbonate solution to stop the reaction. After washing with a large amount of deionized water and drying, a porous surface is obtained. Then the samples were immersed in TFOS for 24 hours for siliconization treatment. Finally, PFD was added to the fluorinated surface and the excess oil layer was removed shortly after to obtain a smooth SLIPS surface.

**Characterization of the dendritic nano-based slippery coating**

**Elemental determination:** The changes in the content of major elements on the surface of PVC catheters before and after modification were analyzed by X-ray photoelectron spectroscopy (XPS) (EscaLab 250Xi, Thermo), including Si 2p peak, Si 2s peak, C 1s peak, N 1s peak and O 1s peak.

**Chemical bond formation/breaking detection:** Fourier infrared spectroscopy (FTIR) (Spectrum TWO, PerkinElmer) was used to analyze the group bonding conditions of different liquids/substances/surfaces.

**Oil test paper test:** The glass surface was modified using solution A (without epoxy resin added), carboxyl silicone oil constructed DNSC-COOH and SLIPS strategy, and the oil test papers were respectively covered on the surface to observe whether there was a free oil layer on the surface.

**Thermal stability test:** Different substances are heated using a thermogravimetric analyzer (Netzsch TG 209) to observe their decomposition.

**Transmittance test:** Ultraviolet-visible transmission spectroscopy was used to investigate the transmittance of the primitive glass (PG) and DNSC-COOH modified surface.

**Hydrophobic performance and slip performance tests:** The contact angle, sliding angle and surface tension of the droplets were measured using the Contact Angle measuring instrument (SDC-350H, China). Select 2 μl of different droplets for contact angle measurement. Select 10 μl of different droplets for sliding angle measurement. Besides, contact angle hysteresis was calculated through the CAH = *θ*_adv_ - *θ*_rec_ formula. In addition, 20 μl of the droplets were selected for macroscopic sliding tests to observe the sliding effect of the droplet on a surface with an inclination of 30° within 3 seconds.

**Droplet fluorescence residue:** Considering that the droplets on the surface of DNSC-COOH can completely slide off, in order to further observe their residue, the droplets on the surface of DNSC-COOH were stained with Rhodamine B (1 μg/ml, Aladdin) and then a slip test was conducted. The fluorescence residue on the modified glass surface was observed under a microscope (Ti2-E, Nikon).

**Strengthening mechanism of the dendritic nano-based slippery coating**

**Particle dispersion:** Amino dendritic nanoparticles and amino spherical nanoparticles were respectively dissolved in DI water and ethyl acetate solution (15g/100ml), and their precipitation and aggregation were observed with the naked eye. Meanwhile, the dispersion/aggregation of the two particles in the ethyl acetate solution was observed by Transmission electron microscope (TEM, FEI Tecnai G2 F20).

**Silicone oil adsorption situation:** Corn oil was used instead of silicone oil and stained with Nile red (0.1mg/ml). Then, the two types of particles were incubated overnight respectively to absorb the corn oil. The excess corn oil was separated by centrifugation at 3000 rpm/min to remove the corn oil that was not captured by the particles. Collect the particles and conduct fluorescence microscope observation and quantitative analysis of fluorescence intensity (Image J).

**Sandpaper abrasion treatment:** Different surfaces were repeatedly rubbed on 1500# sandpaper, with approximately 50 g of weight applied above the sandpaper. The cycle distance was 10 cm, and the number of cycles was 500 times.

**Dynamic blood flow shock treatment:** Select artificial blood to simulate the impact treatment of the blood flow environment (up to 1750 s^-1^ for 15 days). The specific conversion is as follows:

Based on the physiological shear stress of a normal vessel wall (1 ~ 7 Pa), an artificial blood viscosity of 4 mPa·s, and a catheter diameter of 3 mm, the corresponding flow parameters were determined. The conversion process, according to the relevant fluid dynamics principle, is as follows:

$\gamma=\frac{\tau}{\eta}$ (1)

$Q=\frac{\gamma\cdot d}{8}\cdot\pi r^{2}$ (2)

Where $\gamma$ is shear rate, τ is shear force, η is the blood viscosity, $Q$ is volume flow rate, d is catheter diameter and $r$ is catheter radius. The samples were exposed to blood shear rate (Low to 250 s^-1^, the equivalent volume flow rate is about 40 ml/min. Up to 1750 s^-1^, the equivalent volume flow rate is about 280 ml/min).

**Adhesive strength test:** The tape peel tests were carried out in accordance with ASTM D3359-17 standard. In the test, the coating surface was first adhered with 3MTM VHB tape (width ~ 2 cm, adhesion ~ 3000 N/m), and then rolled and pressed on a copper rod (weight ~ 2 kg). The peel test was conducted using a universal testing machine (HZ-1007C) and the adhesive strength was obtained.

**Electrostatic interaction damage treatment:** Three surfaces, DNSC-COOH and DNSC-OH, were immersed in 1 M NaCl, CaCl₂, and FeCl₃ solutions(shear impact: 1750 s⁻¹) for 6 hours and 24 hours, and the changes in their hydrophobic and sliding properties were observed.

**Fluid impact in neutral solution:** Select artificial blood to simulate the impact treatment of the blood flow environment (up to 1750 s^-1^ for 7 days and 15 days), and observe the changes in hydrophobic and sliding properties of the three surfaces (DNSC-COOH, DNSC-OH and DNSC-OCH_3_).

**Observation of cross-sectional morphology:** scanning electron microscope (SEM) was used to observe the changes in the cross-sectional morphology of DNSC-COOH after different concentrations of aminated dendritic nanoparticles and under fluid impacts for 7 days and 15 days.

**Anti-adhesion Properties of the dendritic nano-based slippery coating**

**Anti-protein adhesion test**

To evaluate the anti-adhesion efficacy, fluorescein-labeled bovine serum albumin (FITC-BSA, Solarbio) and fluorescein-conjugated fibrinogen (Fg, Solarbio) were employed as model proteins. Their protein concentrations were approximately 5.12 mg/ml and 5 mg/ml, respectively. Both the DNSC-COOH modified DNSC-MC and the unmodified PC (as a control) samples were incubated with these protein solutions in 96-well plates. This incubation was conducted statically at 37 °C for 24 hours in darkness. Subsequently, each sample was gently rinsed with PBS buffer (0.01 M, pH 7.2) to eliminate any unbound protein. Protein adhesion was then characterized using a Nikon Ti2-E fluorescence microscope after identical exposure times. Image J software was utilized for the quantitative statistical analysis of the relative fluorescence intensity, which indicated the amount of protein adsorbed on the sample surfaces.

**Anti-bacteria adhesion test**

To assess the antibacterial adhesion properties, *E. coli* (ATCC25922) and *S. aureus* (ATCC6538), obtained from Shanghai LuWei Microbial Tech. CO. LTD, were utilized as model bacteria. The DNSC-MC samples and a bacterial suspension (100 μl, 1×10⁹–10¹⁰ CFU/ml) were co-incubated in 96-well plates at 37 °C for 24 hours. Following incubation, the samples were stained with 10 μg/ml SYTO-9 green fluorescent dye (Thermo Fisher Scientific) and subsequently rinsed once with PBS. Bacterial adhesion on the surfaces was immediately observed under a fluorescence microscope under consistent exposure settings. Image J software was employed to quantify the relative fluorescence intensity, which corresponds to the number of adherent bacteria.

**Anti-cells adhesion test**

In order to study the influence of cells adhesion behavior and activity, we selected NIH 3T3 (CL-0171, Wuhan Procell) and Raw 264.7 (CL-0190, Wuhan Procell) as cell models for exploration. The cells were seeded into 96-well plates, which contained either DNSC-MC samples or served as blank controls, and incubated for 24 hours at 37 °C. After the incubation period, cell viability was assessed by double staining with fluorescent dyes: Calcein AM (10 μg/ml, Thermo Fisher Scientific) to label live cells and Propidium Iodide (10 μg/ml, Thermo Fisher Scientific) to label dead cells. The stained cells were imaged under a fluorescence microscope using consistent exposure times for all groups. Finally, the numbers of live and dead cells on the surfaces were quantified using Image J software.

**Test of protein activity**

The impact of DNSC-COOH on enzymatic activity was evaluated using glucose oxidase (GOD, Aladin). DNSC-MC samples were incubated with a 0.1 mg/mL GOD solution (in PBS) at 37 °C for 24 hours. After incubation, the remaining enzymatic activity was measured using a commercial GOD activity detection kit (Solarbio). For comparison, a blank control (GOD solution without sample) and a heat-inactivated control (GOD heated at 90 °C for 0.5 hours) were analyzed respectively.

**Test of bacteria activity**

The antibacterial effect of DNSC-COOH was investigated by co-culturing *E. coli* and *S. aureus* with DNSC-MC samples for 24 hours at 37 °C. Following incubation, bacterial viability was assessed using a two-color fluorescence assay: SYTO-9 (10 μg/ml, Thermo Fisher Scientific) to stain live cells and Propidium Iodide (10 μg/ml) to stain dead cells. The bottom of a blank 96-well plate served as the control. The stained bacteria were visualized under a fluorescence microscope at consistent exposure times, and the viability was quantified by calculating the percentage of live and dead cells.

**Test of cells activity**

The cytocompatibility of DNSC-MC samples was evaluated using NIH 3T3 and Raw 264.7 cell lines. Cells were seeded into 96-well plates, with experimental wells containing DNSC-MC samples and control wells being blank. After a 24-hour incubation at 37 °C, cell viability was assessed by double staining with Calcein AM (10 μg/ml, for live cells) and Propidium Iodide (10 μg/ml, for dead cells). Fluorescence images were captured under a microscope using consistent exposure times. The numbers of live and dead cells adherent to the sample surfaces were subsequently quantified using Image J software.

**The growth condition of the cytoskeleton**

After 24 hours of cell climbing, when the density reached 50% confluence, the cells were washed twice and fixed for 10 minutes using a 4% formaldehyde solution dissolved in PBS. Then, at room temperature, washed the cells 2 to 3 times with PBS, each time for 10 minutes. Permeated with 0.5%Triton X-100 (Solarbio) solution for 5 minutes; Washed the cells 2 to 3 times, each time for 10 minutes. Took 200 μl of the prepared FITC-labeled Phalloidin (Solarbio) working solution, cover the cells on the cover slip, incubated at 37 ° C in the dark for 120 minutes and washed the cells three times. The nuclei were stained with 200 μl of 4',6-diamidino-2-phenylindole (DAPI, 10 ug/ml, Solarbio) solution for 10 minutes. The cover slips were washed with PBS and then sealed with antifluorescence quenchers. Fluorescence observation under a fluorescence microscope.

**Antithrombotic effect and *in vivo* biosafety** **of the dendritic nano-based slippery coating**

**Test of antithrombosis properties *in vitro and vivo*:** We investigated the *in vivo* antithrombotic and anti-inflammatory properties of DNSC-COOH using New Zealand rabbits extracorporeal shunt model (male, 3.0-3.5 kg). All animal procedures for the *in vitro* and *in vivo* thrombogenicity tests were performed in strict compliance with local ethical guidelines and were approved by the Laboratory Animal Welfare and Ethics Committee of Zhujiang Hospital, Southern Medical University (Approval Nos. LAEC-2024-036FS and LAEC-2024-036FS2). A 0.5-meter-long DNSC-MC and Commercial sterile PVC catheter (PC) were integrated into an arteriovenous shunt model in rabbits. After 2 hours of circulation, the catheters were observed the thrombosis in the catheter loop, and measured the weight of thrombus, catheter occlusion rate and blood flow velocity, and comprehensively evaluate its antithrombotic performance. Catheter patency and blood flow rate were quantitatively analyzed using computer-assisted image analysis. Furthermore, blood samples were collected at predetermined intervals (0, 10, 30, 60 minutes) to interrogate the systemic response through biochemical analysis. Key biomarkers included inflammatory markers (WBC, CRP, TNF-α, IL-6, IL-10), coagulation parameters (TAT, PIC, TM, ALB and PLT) and potential toxicity markers (ALT, Scr).

***In vitro* whole blood closed circulation test:** The DNSC-MC and PC were also connected in series to the circuit, with fresh whole blood labeled with fluorescent fibrinogen (Fg, 5 v/v%) used for circulation experiments. After 2-hour circulation and were washed with PBS for 2 times, the fibronectin adhesion on the catheter surface was quantitatively observed using a fluorescence microscope, while platelet adhesion morphology was analyzed through scanning electron microscopy (SEM) to compare surface platelet adhesion.

**Hemolysis test:** Centrifuge the New Zealand rabbit blood at 1000 rpm/min for 10 minutes and remove the supernatant. Subsequently, the cells were repeatedly washed 2 to 3 times with 0.9% NaCl and resuspended to obtain a 5% red blood cell suspension. 100 μl of the red blood cell suspension was added to 900 μl of 0.9% NaCl solution containing DNSC-MC sample, DI water (positive control), and 0.9% NaCl solution (negative control), and incubated for 1 hour. Centrifuge and take the supernatant to read the absorbance of the supernatant at 540 nm. Each experiment was repeated three times. The formula for calculating hemolysis rate: [(OD_DNSC-MC_-OD_negative control_)/(OD_positive control_ - OD_negative control_)]× 100%.

**Subcutaneous implantation toxicity test:** The *in vivo* biocompatibility of DNSC-COOH was further assessed by implanting DNSC-MC samples into the subcutaneous tissue of SD rats for 15 days. The host response was evaluated based on acute inflammation, and early-stage foreign body reaction. The original antibacterial catheter was used as a control for comparison.

**Long-term advantages in anti-biological adhesion and anti-thrombosis** **of Nano-Based “Solid-Like” Slippery Composite Coating**

**Anti-biological substance adhesion test:** DNSC-COOH modified catheter (DNSC-MC) samples and LIS modified catheter samples were exposed to low physiological shear rates (up to 250 s^-1^) in a fluid environment (artificial blood) using a microfluidic device (Kamoer), and could be treated for 7, 14, and 30 days, respectively. Exposure to a high physiological shear rate (up to 1750 s^-1^) for 15 days. The anti-adhesion effect of the sample surface after being impacted by fluids under different conditions was verified by the same test methods for anti-protein/cell/bacterial adhesion and anti-thrombosis, staining methods and quantitative analysis methods.

**Environmental stability test:** They were treated under high pressure conditions of 0.1 MPa and 120 °C for 30 minutes respectively, irradiated in an environment with ultraviolet intensity of 70 μW/cm² for 60 minutes, and treated with ultrasonic frequency of 20 kHz for 30 minutes. Besides, the vessel wall friction is usually 1 ~ 7 Pa and the friction area of the target catheter is 0.000942 cm^2^ (catheter diameter 3 mm, length 10 cm). The surface of DNSC-COOH is repeatedly rubbed on 1500# sandpaper, applying a weight of about 1 g (equivalent to 10 Pa of the vessel wall friction, 1000 times) for a cycle distance of 5 cm. Finally, immersed in PH=1, PH=5.5, PH=7.5 and 0.9% NaCl solution for 30 days. The changes in its anti-protein adhesion performance and sliding performance before and after treatment were systematically evaluated.

**Supplementary Figures**

*
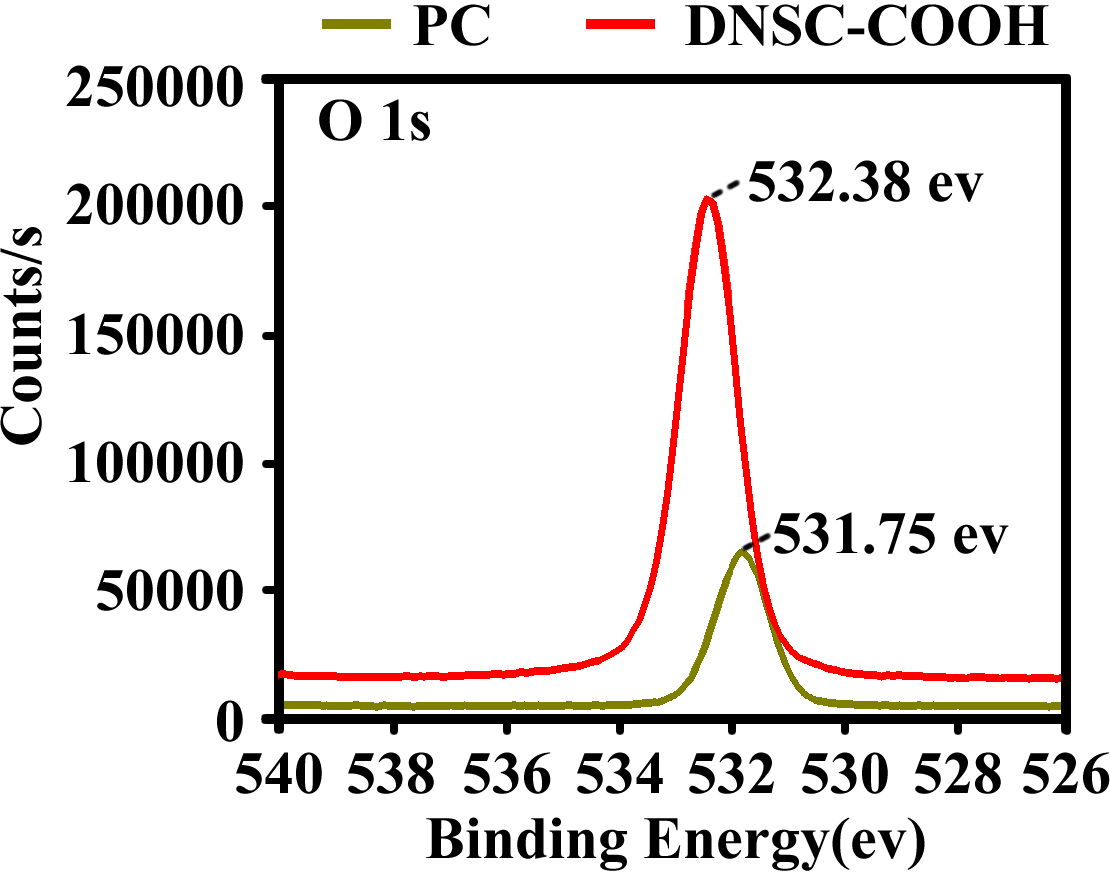
*

**Figure S1. O 1s XPS spectrum.** The O 1s main peak of DNSC-COOH system increased from 531.75 ev to 532.38 ev, suggesting that oxygen atoms might have interfuncted with stronger groups.


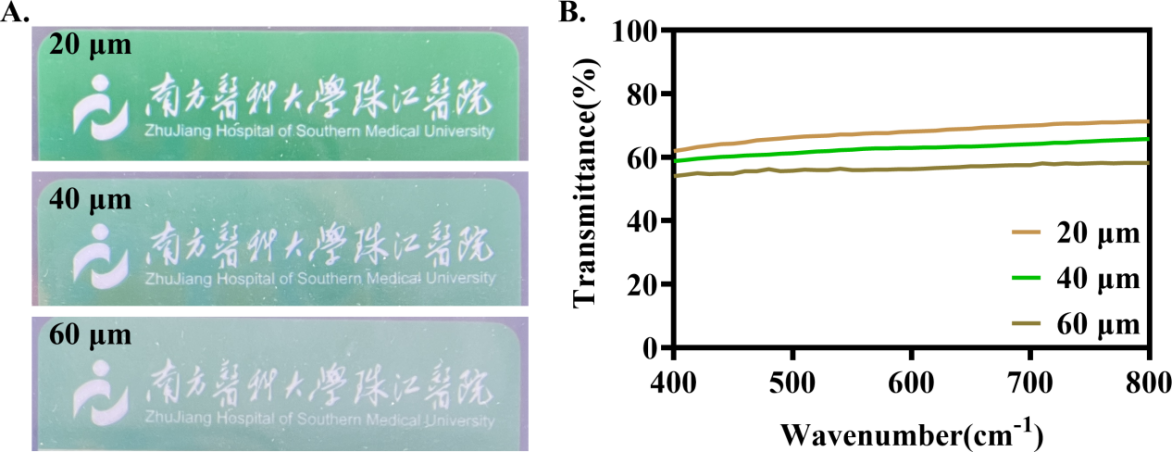


**Figure S2. Explore the influence of coating thickness on the light transmission performance of glass.** A) As the thickness (20, 40, 60 μm) of DNSC-COOH continues to increase, its clarity gradually decreases, resulting in visual differences in the covered content. B) The transmittance also correspondingly drops from about 80% to about 54%, and the clarity is negatively correlated with the film thickness.


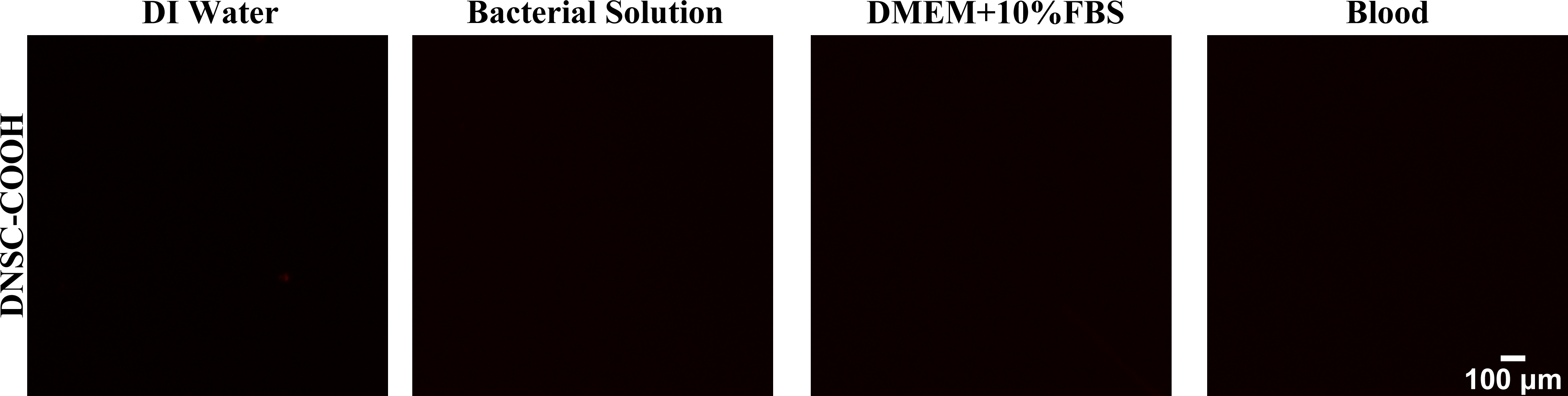


**Figure S3. The residual condition of liquid droplets on the surface of DNSC-COOH.** The sliding performance test was conducted by using Rhodamine B fluorescently labeled droplets on the surface of DNSC-COOH. The fluorescence images show that there was no residual fluorescence in the droplet sliding path, highlighting the excellent sliding performance of DNSC-COOH.


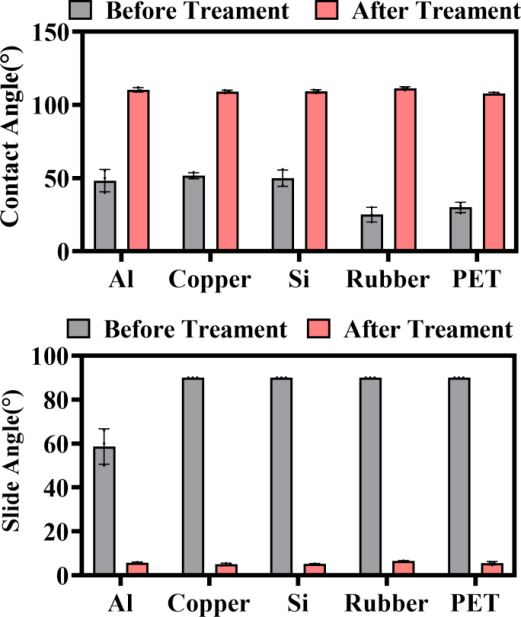


**Figure S4. The influence of DNSC-COOH on the hydrophobic and sliding properties of different substrates.** After modifying the different substrates with DNSC-COOH, the contact Angle increased significantly and the sliding angle decreased significantly. This indicates that DNSC-COOH endows the substrates with excellent hydrophobic and sliding properties.


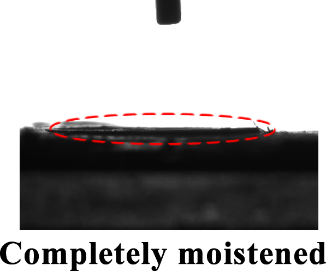


**Figure S5.** When the mass ratio of nanoparticles to silicone oil reaches 300:100, the surface shows a phenomenon of complete wetting of water droplets due to the presence of a large number of loose particles, and CA and SA cannot be measured.


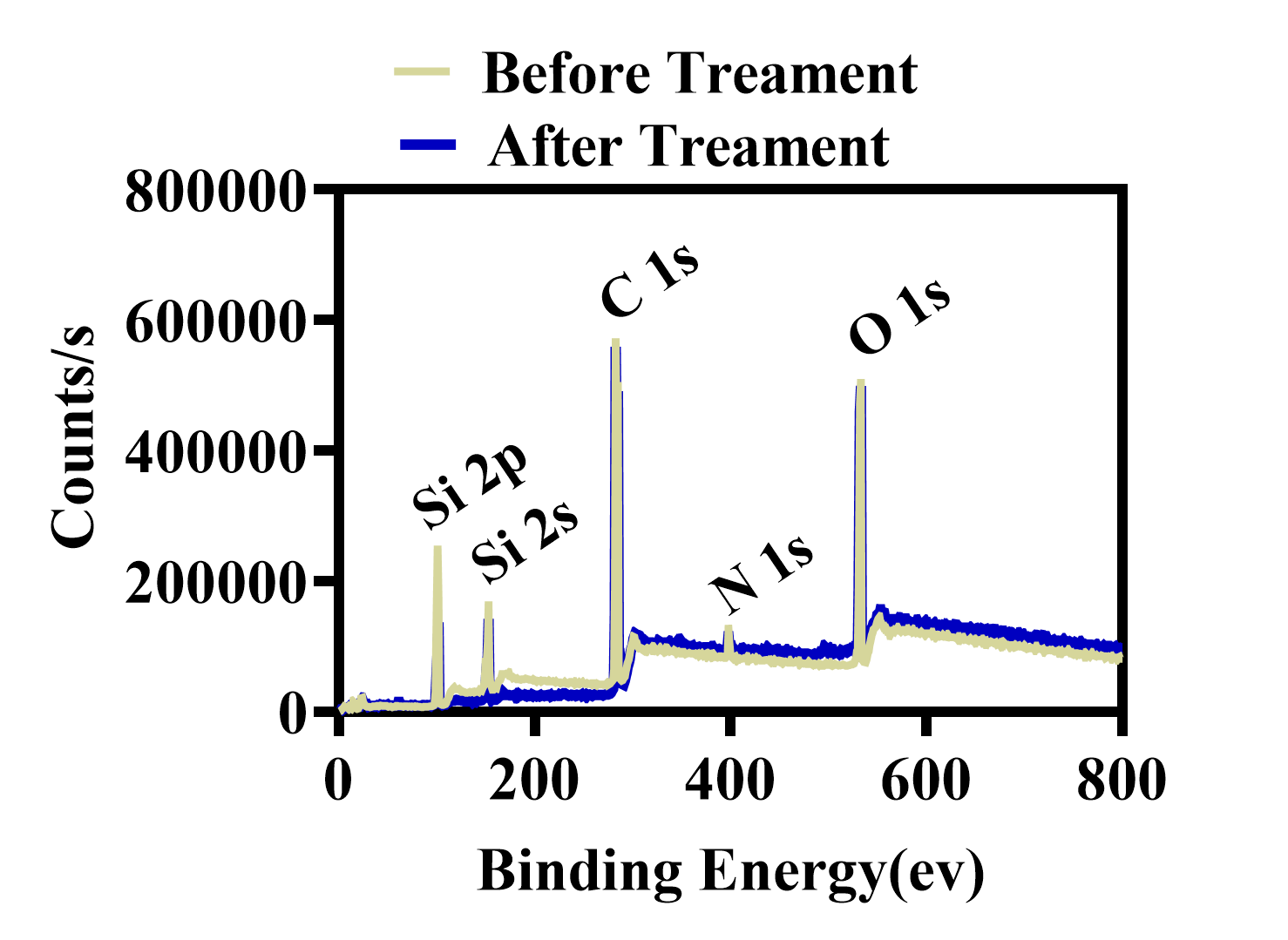


**Figure S6. XPS data on the surface elemental composition of the DNSC-COOH before and after 15 days of exposure to neutral high-speed blood flow.** As can be seen from the figure, the content of the main elements did not change significantly before and after the treatment.


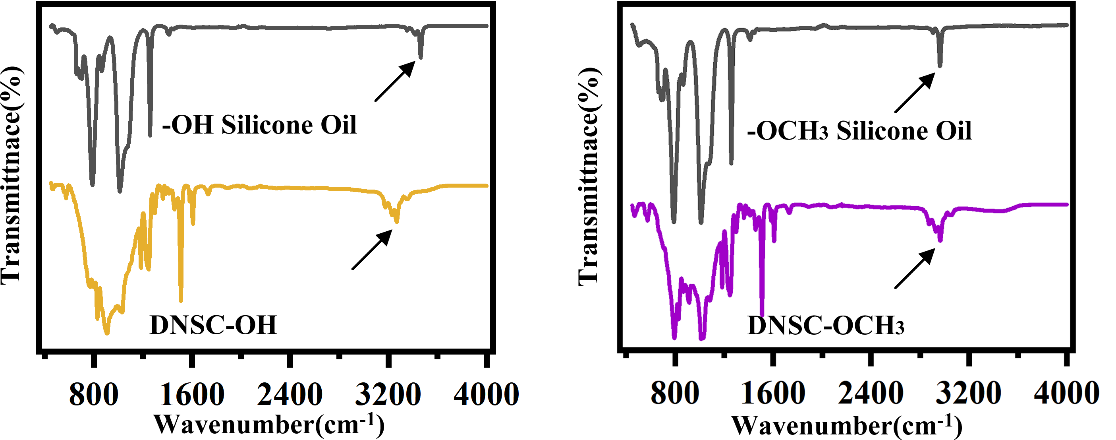


**Figure S7. Results of the FTIR spectrum analyses of DNSC-OH and DNSC-OCH_3_.** In the DNSC-OH system, the O-H expansion vibration absorption peak indicated that a hydrogen bond had formed between the nanoparticles and the -OH silicone oil, and its stability depends on the effect of the hydrogen bond. However, in the DNSC-OCH_3_ system, the absorption peaks of the relevant groups did not move, indicating that no related hydrogen bonds were formed in the DNSC-OCH_3_ system.


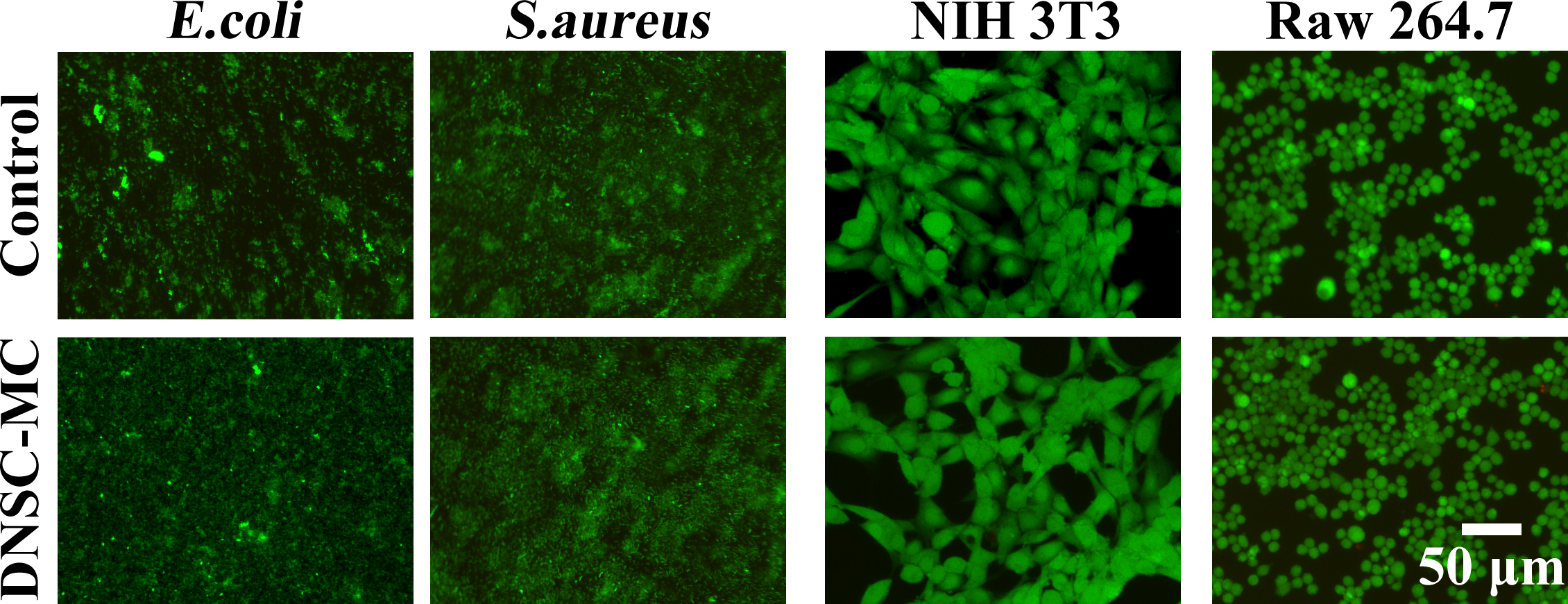


**Figure S8. Activity test.** Compared with the Control group, DNSC-MC did not cause toxic effects on bacteria and cells to lead to their death. This also means that DNSC-COOH does not kill the active substances but rather relies on its surface “slippery” advantage to counteract the adhesion of biological substances.


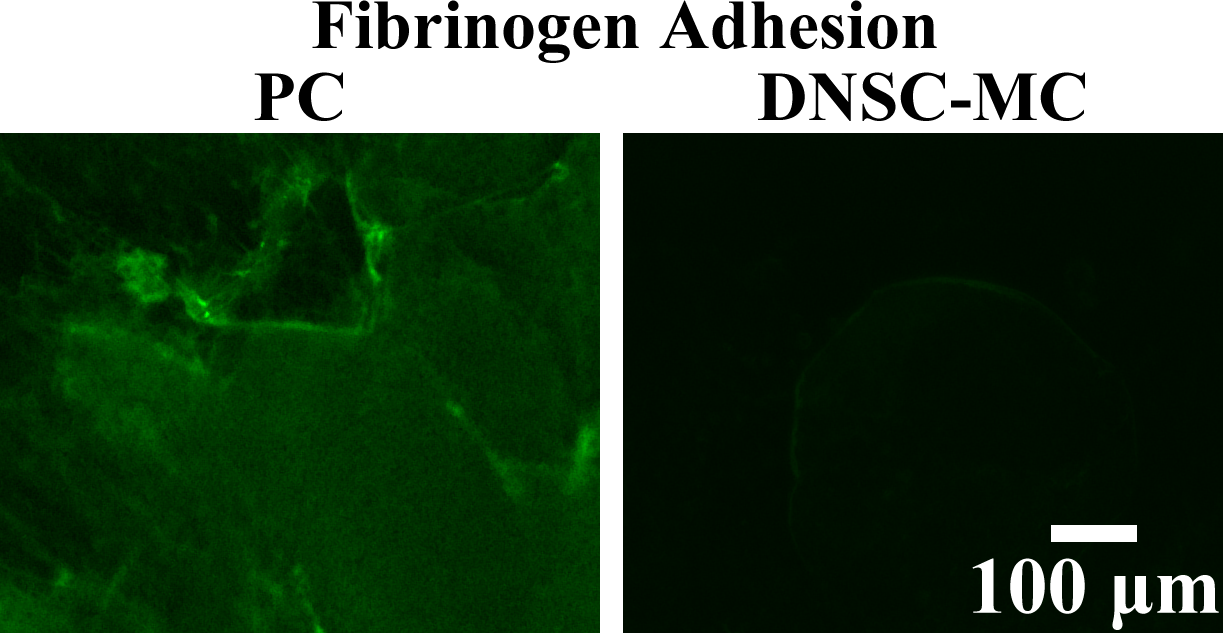


**Figure S9. Fibrinogen adhesion.** Fresh rabbit blood was labeled with Fg, and the adhesion of fibrin on the tube wall was observed after 2 hours of cardiopulmonary circulation.


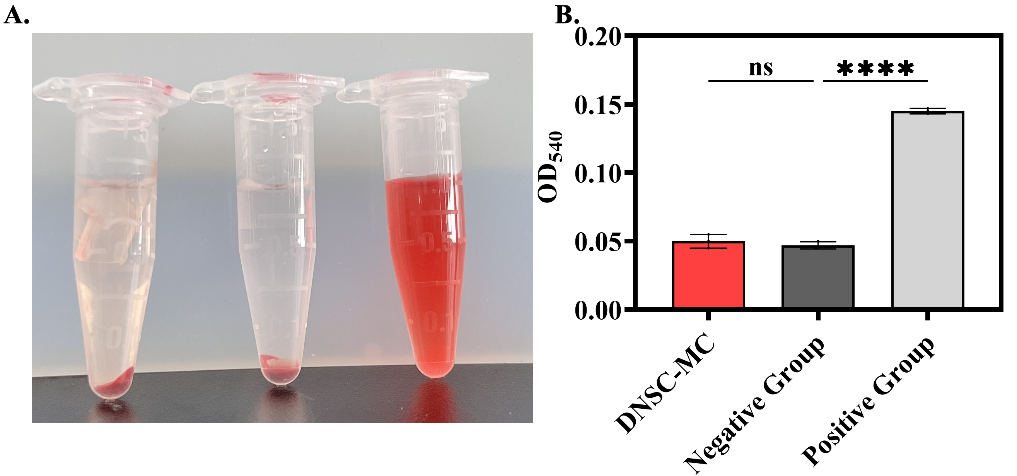


**Figure S10. Hemolysis test results.** A hemolysis rate of 3.1% was measured for the DNSC-MC group following a 1-hour incubation with red blood cells, confirming its excellent blood compatibility (<5%).


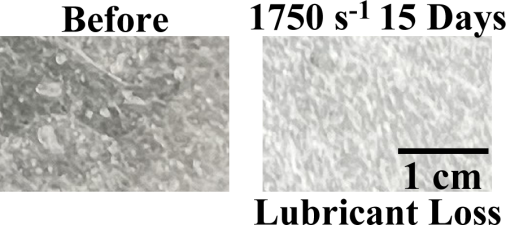


**Figure S11. Comparison of lubrication layer loss on LIS.** After being affected by the high shear rate environment of artificial blood (1750 s^-1^ for 7 days), the free lubricating layer on the surface of LIS almost disappeared.


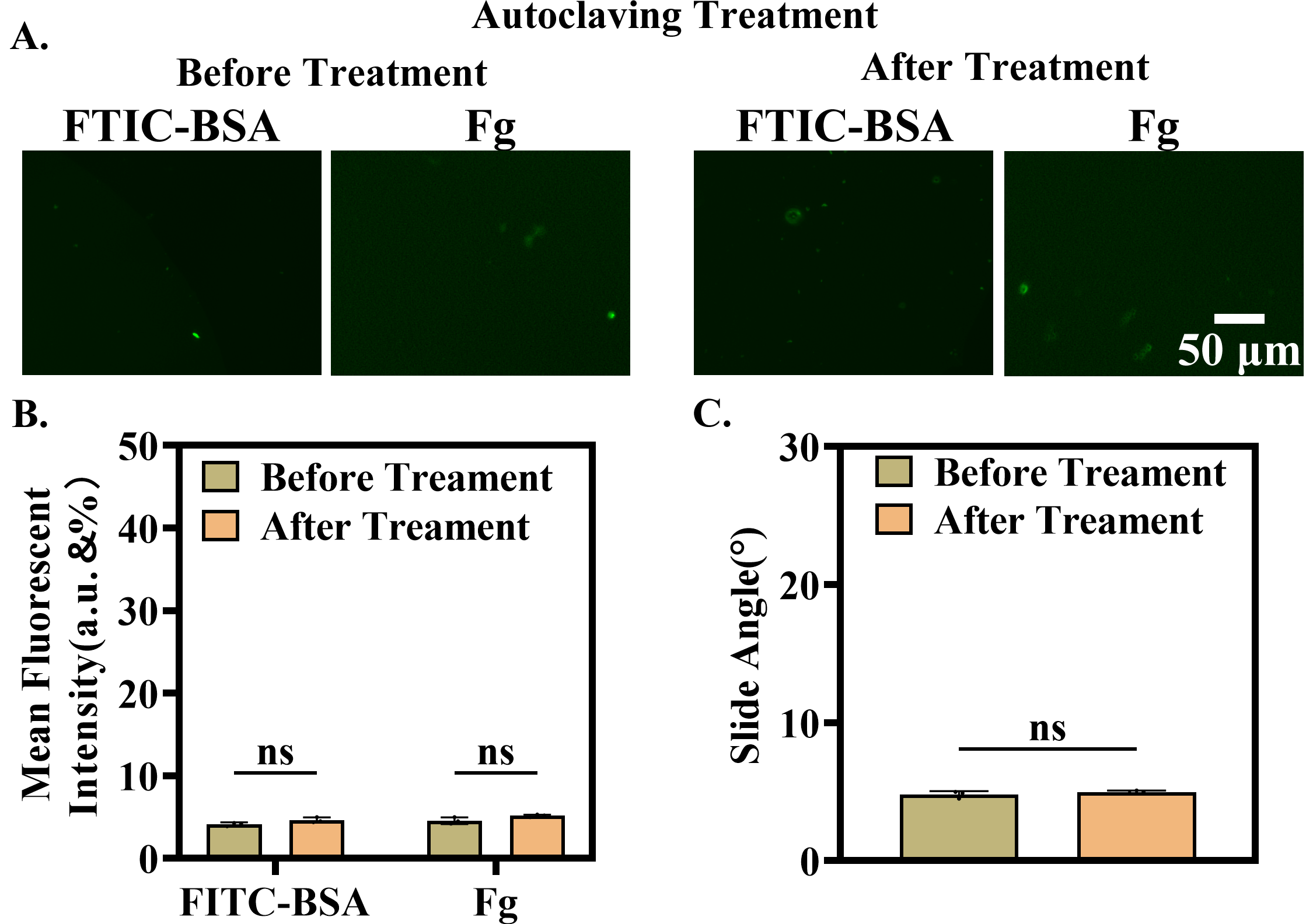


**Figure S12.** A) The fluorescence representative diagram of anti-protein adhesion on the surface of DNSC-MC after autoclaving treatment, B) quantitative analysis and C) the change of sliding Angle. Error bar represents the mean ± SD. n = 3, averaged.

**
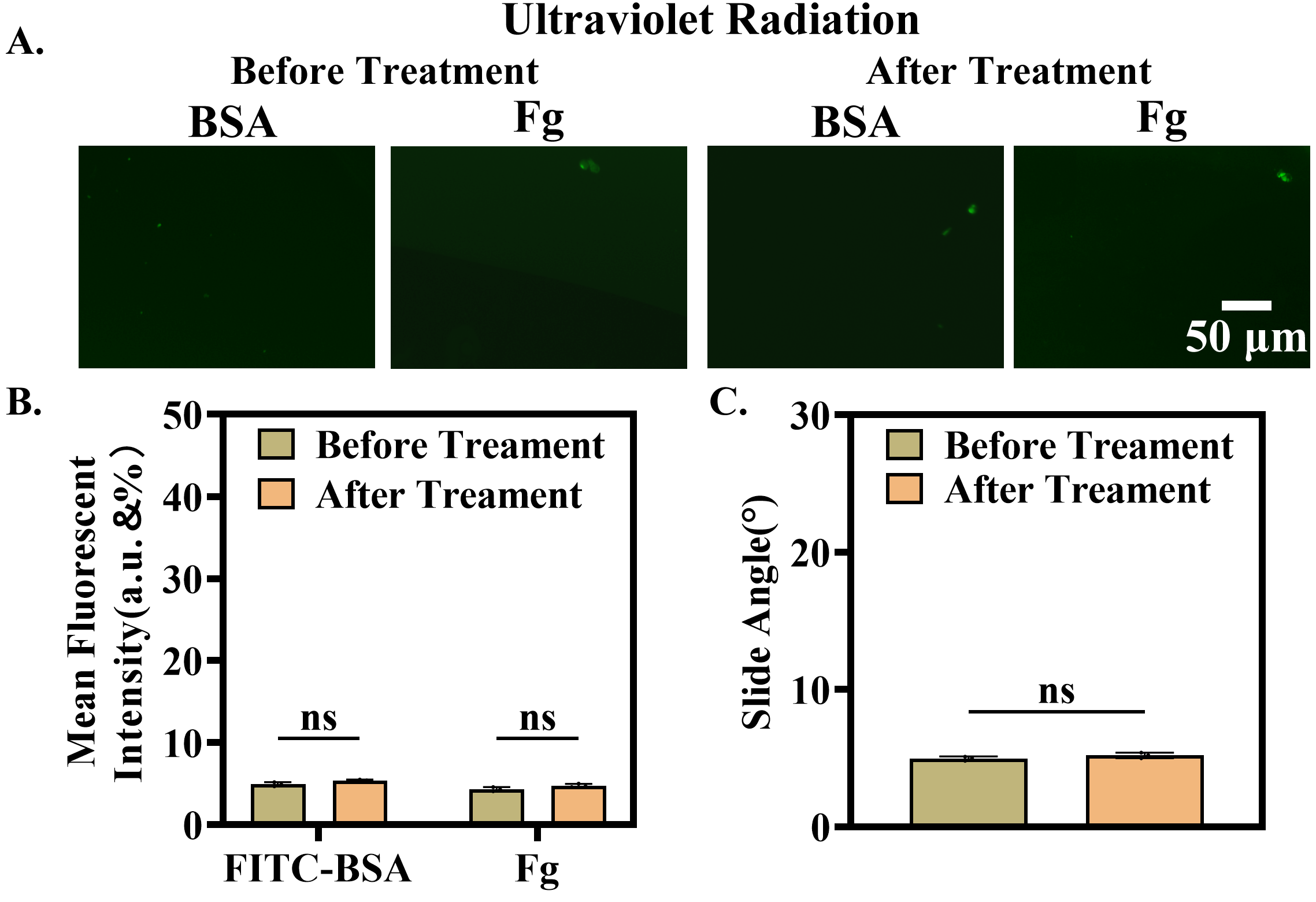
**

**Figure S13.** A) The fluorescence representative diagram of anti-protein adhesion on the surface of DNSC-MC after ultraviolet radiation treatment, B) quantitative analysis and C) the change of sliding Angle. Error bar represents the mean ± SD. n = 3, averaged.

**
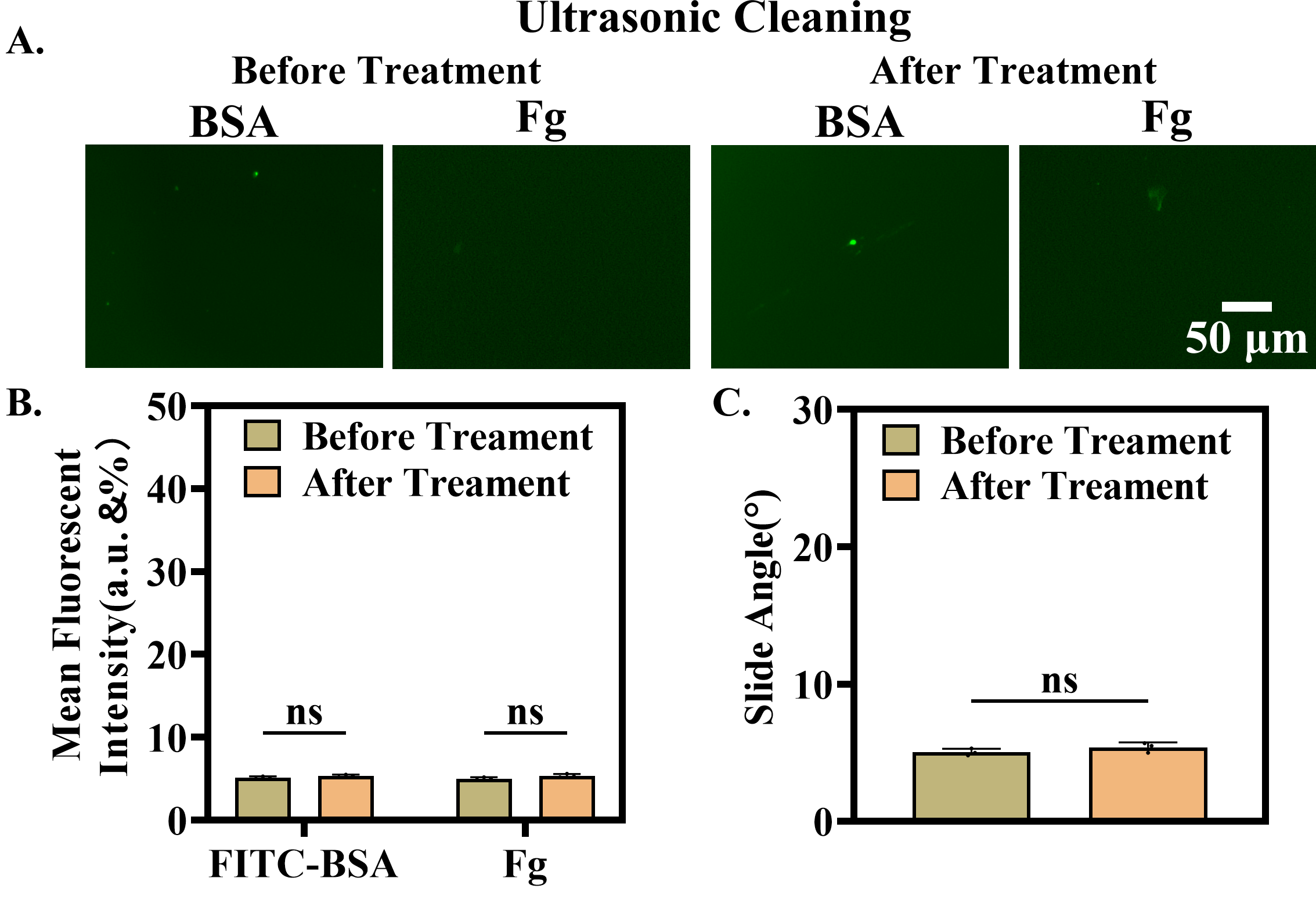
**

**Figure S14.** A) The fluorescence representative diagram of anti-protein adhesion on the surface of DNSC-MC after ultrasonic cleaning treatment, B) quantitative analysis and C) the change of sliding Angle. Error bar represents the mean ± SD. n = 3, averaged.

**
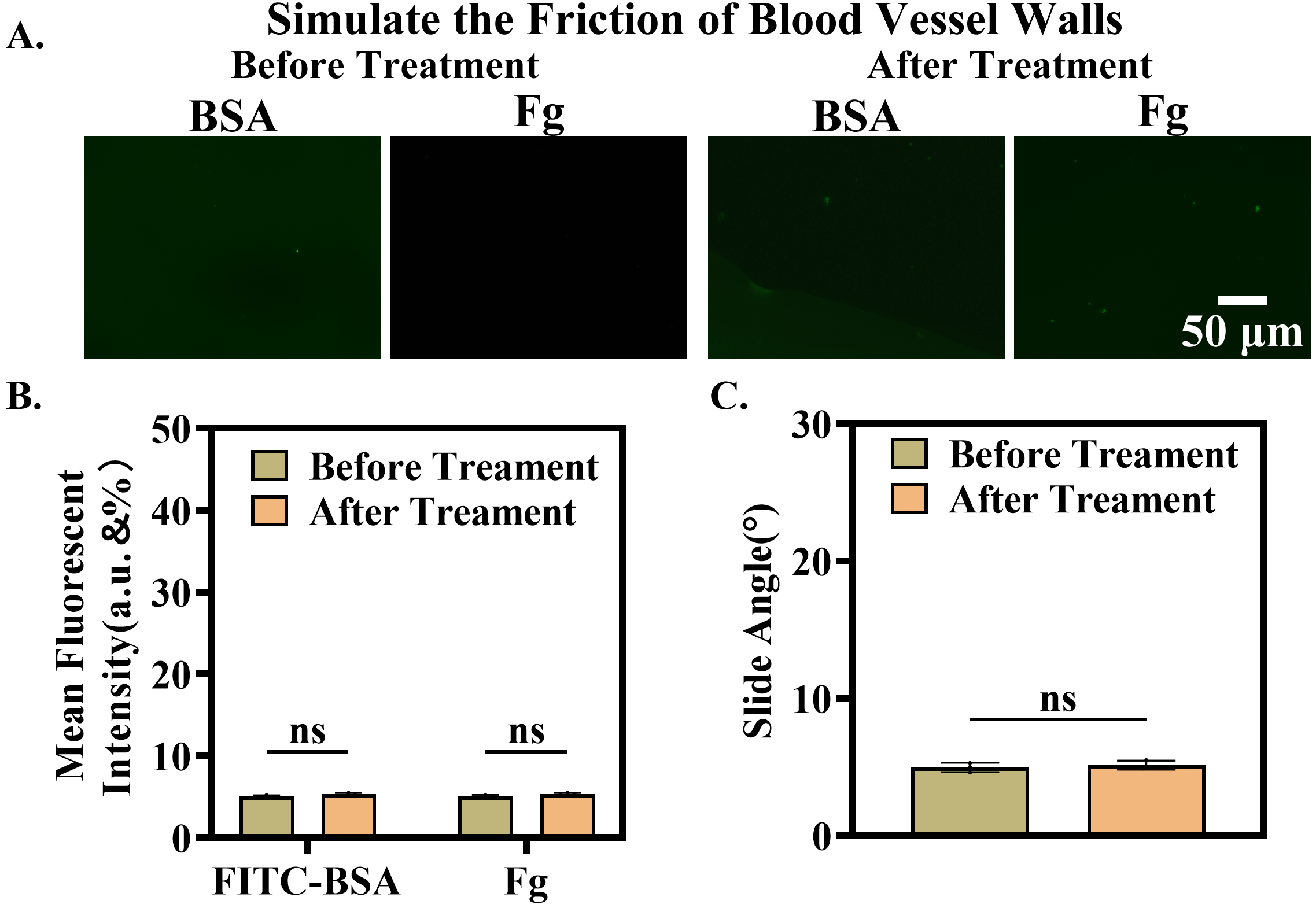
**

**Figure S15.** A) The fluorescence representative diagram of anti-protein adhesion on the surface of DNSC-MC after simulated vascular wall friction treatment, B) quantitative analysis and C) the change of sliding Angle. Error bar represents the mean ± SD. n = 3, averaged.

**
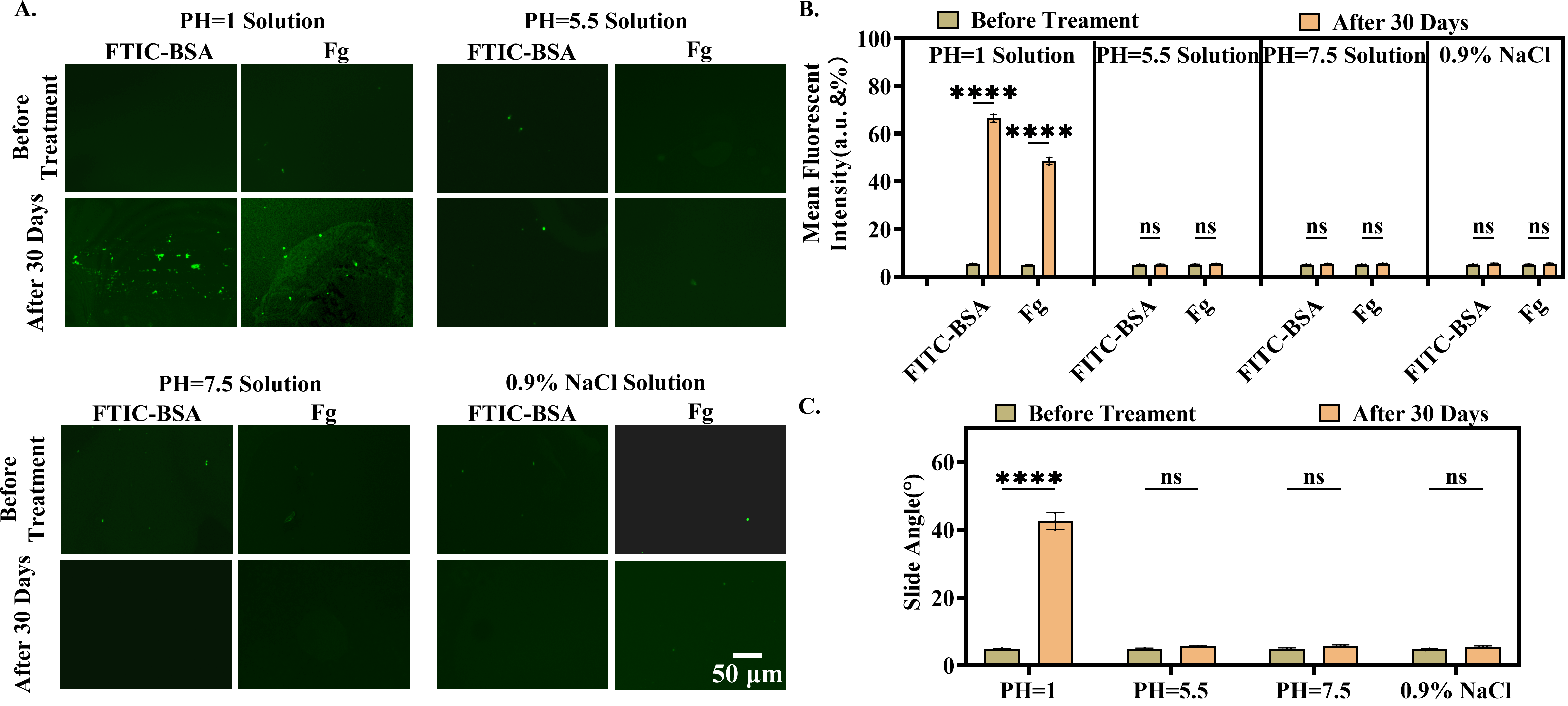
**

**Figure S16.** A) The fluorescence representative diagram of anti-protein adhesion on the surface of DNSC-MC after immersion in different solutions simulating physiological environments, B) quantitative analysis and C) the change of sliding angle. Error bar represents the mean ± SD. n = 3, averaged.
